# Optimal timing of influenza vaccine during pregnancy: A systematic review and meta‐analysis

**DOI:** 10.1111/irv.12649

**Published:** 2019-06-05

**Authors:** Will Cuningham, Nicholas Geard, James E. Fielding, Sabine Braat, Shabir A. Madhi, Marta C. Nunes, Lisa M. Christian, Shin‐Yu Lin, Chien‐Nan Lee, Koushi Yamaguchi, Hans Bisgaard, Bo Chawes, An‐Shine Chao, Geraldine Blanchard‐Rohner, Elizabeth P. Schlaudecker, Barbra M. Fisher, Jodie McVernon, Robert Moss

**Affiliations:** ^1^ Menzies School of Health Research Charles Darwin University Casuarina Northern Territory Australia; ^2^ Victorian Infectious Diseases Reference Laboratory, Epidemiology Unit, Peter Doherty Institute for Infection and Immunity The Royal Melbourne Hospital and The University of Melbourne Melbourne Victoria Australia; ^3^ Centre for Epidemiology and Biostatistics Melbourne School of Population and Global Health The University of Melbourne Melbourne Victoria Australia; ^4^ Computing and Information Systems, Melbourne School of Engineering The University of Melbourne Melbourne Victoria Australia; ^5^ Melbourne Clinical and Translational Sciences (MCATS) Platform, Melbourne School of Population and Global Health The University of Melbourne Melbourne Victoria Australia; ^6^ Medical Research Council: Respiratory and Meningeal Pathogens Research Unit, Faculty of Health Science University of the Witwatersrand Johannesburg South Africa; ^7^ Department of Science/National Research Foundation: Vaccine Preventable Diseases, Faculty of Health Science University of the Witwatersrand Johannesburg South Africa; ^8^ The Department of Psychiatry & Behavioral Health and The Institute for Behavioral Medicine Research The Ohio State University Wexner Medical Center Columbus Ohio; ^9^ Department of OBS & GYN National Taiwan University Hospital Taipei Taiwan; ^10^ Division of Immunology and Microbiology, Center of Maternal‐Fetal, Neonatal and Reproductive Medicine National Center for Child Health and Development Tokyo Japan; ^11^ COPSAC, Copenhagen Prospective Studies on Asthma in Childhood, Herlev and Gentofte Hospital University of Copenhagen Copenhagen Denmark; ^12^ Department of Obstetrics & Gynecology Chang Gung Memorial Hospital & Chang Gung University Taipei Taiwan; ^13^ Department of Pediatrics Children’s Hospital of Geneva, University Hospitals of Geneva and Faculty of Medicine Geneva Switzerland; ^14^ Division of Infectious Diseases, Global Health Center Cincinnati Children’s Hospital Medical Center Cincinnati Ohio; ^15^ Department of Obstetrics and Gynecology, Section of Maternal‐Fetal Medicine University of Colorado School of Medicine Aurora Colorado; ^16^ Murdoch Children’s Research Institute The Royal Children’s Hospital Melbourne Victoria Australia

**Keywords:** immunogenicity, influenza, pregnancy, timing, trimester, vaccination

## Abstract

**Background:**

Pregnant women have an elevated risk of illness and hospitalisation from influenza. Pregnant women are recommended to be prioritised for influenza vaccination during any stage of pregnancy. The risk of seasonal influenza varies substantially throughout the year in temperate climates; however, there is limited knowledge of how vaccination timing during pregnancy impacts the benefits received by the mother and foetus.

**Objectives:**

To compare antenatal vaccination timing with regard to influenza vaccine immunogenicity during pregnancy and transplacental transfer to their newborns.

**Methods:**

Studies were eligible for inclusion if immunogenicity to influenza vaccine was evaluated in women stratified by trimester of pregnancy. Haemagglutination inhibition (HI) titres, stratified by trimester of vaccination, had to be measured at either pre‐vaccination and within one month post‐vaccination, post‐vaccination and at delivery in the mother, or in cord/newborn blood. Authors searched PubMed, Scopus, Web of Science and EMBASE databases from inception until June 2016 and authors of identified studies were contacted for additional data. Extracted data were tabulated and summarised via random‐effect meta‐analyses and qualitative methods.

**Results:**

Sixteen studies met the inclusion criteria. Meta‐analyses found that compared with women vaccinated in an earlier trimester, those vaccinated in a later trimester had a greater fold increase in HI titres (1.33‐ to 1.96‐fold) and higher HI titres in cord/newborn blood (1.21‐ to 1.64‐fold).

**Conclusions:**

This review provides comparative analysis of the effect of vaccination timing on maternal immunogenicity and protection of the infant that is informative and relevant to current vaccine scheduling for pregnant women.

## 1 INTRODUCTION

Pregnant women have a particularly high risk of illness and hospitalisation from influenza. During pregnancy, women experience physiological changes in their cardiopulmonary and immunological systems.^1^, ^2^ An increase in oxygen consumption, a decrease in lung capacity and the suppression of cell‐mediated immunity to tolerate the growth of a genetically foreign foetus all increase pregnant women's susceptibility to infectious diseases and respiratory pathogens such as influenza.^3^, ^4^, ^5^ The risks of hospitalisation and complications for respiratory illness during the influenza season are higher for pregnant women and increase by trimester.^6^, ^7^ Furthermore, pregnant women infected with influenza might be more likely to have adverse birth outcomes.^3^, ^8^, ^9^, ^10^

Vaccination is the most effective preventative measure against influenza infection,^8^, ^11^ and influenza vaccines have been recommended for use in pregnant women for many decades.^12^ The safety, effectiveness and immunogenicity of influenza virus vaccines during pregnancy have been studied extensively, and there is good evidence to support current vaccination recommendations.^13^, ^14^, ^15^, ^16^ The World Health Organization and the US Centers for Disease Control and Prevention prioritise pregnant women for vaccination,^17^ and the Advisory Committee on Immunization Practices and the American College of Obstetricians and Gynecologists have recommended the inactivated seasonal influenza vaccine to women in any trimester since 2004.^17^, ^18^ Evidence of additional benefits of maternal influenza vaccination, such as the protection of young infants via placental transfer of protective antibodies to the foetus, provides further support for antenatal vaccination.^19^, ^20^ Moreover, the interruption of influenza virus transmission by vaccinating the mother, together with transplacental transfer of vaccine‐associated antibody, also reduces the risk of infection for infants 3‐4 months old (before direct vaccination is possible).^21^

Despite the heightened risk of influenza illness in pregnant women and benefits of vaccination, vaccination coverage rates in this population remain suboptimal. In recent years, coverage rates in the United States and Australia have ranged from 20%‐50%.^22^, ^23^, ^24^, ^25^ Surveys have attributed these low vaccine uptake rates in part to distrust in the healthcare system, unawareness of the risks of influenza infection during pregnancy, concerns about vaccine safety for the foetus and lack of encouragement from healthcare professionals.^8^, ^22^, ^23^, ^24^

Recommendations for the timing of influenza vaccination during pregnancy have varied. Although immunisation is now recommended for women at any stage of pregnancy,^26^ the timing of vaccination to optimise benefit to the mother and their infants is not well established. A structured analysis of the optimal timing of influenza vaccination during pregnancy would inform specific scheduling recommendations to pregnant women and maximise the benefit received by vaccination.

Previous reviews of antenatal influenza vaccination have reported limited and mixed evidence on the association between influenza vaccination, influenza infection and adverse birth outcomes, and have not examined the relationship between vaccination timing and immunogenicity.^21^, ^27^, ^28^, ^29^, ^30^, ^31^, ^32^ This systematic review examined whether the timing of influenza vaccination during pregnancy affects the immunogenicity of the vaccine in the mother and transplacental transfer of antibody to the newborn.

## 2 METHODS

This review was conducted in accordance with the PRISMA^33^ checklist (Table S1).

### 2.1 Eligibility criteria

Our population of interest was women vaccinated during pregnancy with a seasonal or pandemic vaccine. Each study was required to include women vaccinated in different trimesters to enable comparison of outcomes between trimesters. All study designs were eligible for inclusion.

The primary outcome was geometric mean titre (GMT) measured by haemagglutination inhibition (HI) assays. Geometric mean titres had to be measured either at (a) both pre‐vaccination and within one month post‐vaccination, (b) both post‐vaccination and delivery in the mother, or (c) delivery in cord blood or newborn blood (hereafter referred to as cord blood for simplicity). If reported in the included studies, seroprotection (HI titre of ≥1:40) and seroconversion (≥4‐fold increase in HI titre) rates were also discussed.^34^ We did not assess vaccine safety or adverse outcomes in the mother or newborn.

### 2.2 Search strategy

A systematic search of PubMed, Scopus, Web of Science and EMBASE was conducted across records published in English from inception to June 2016 using the following search terms with no field restrictions:

1. [influenza OR flu OR H1N1] AND.
2. [vaccin* OR immuni*] AND.
3. [mother OR pregnan* OR maternal OR antenatal OR trimester] AND.
4. [season* OR pandemic OR time OR timing OR monovalent OR inactivated OR TIV OR trivalent OR IIV].

**Figure 1:**
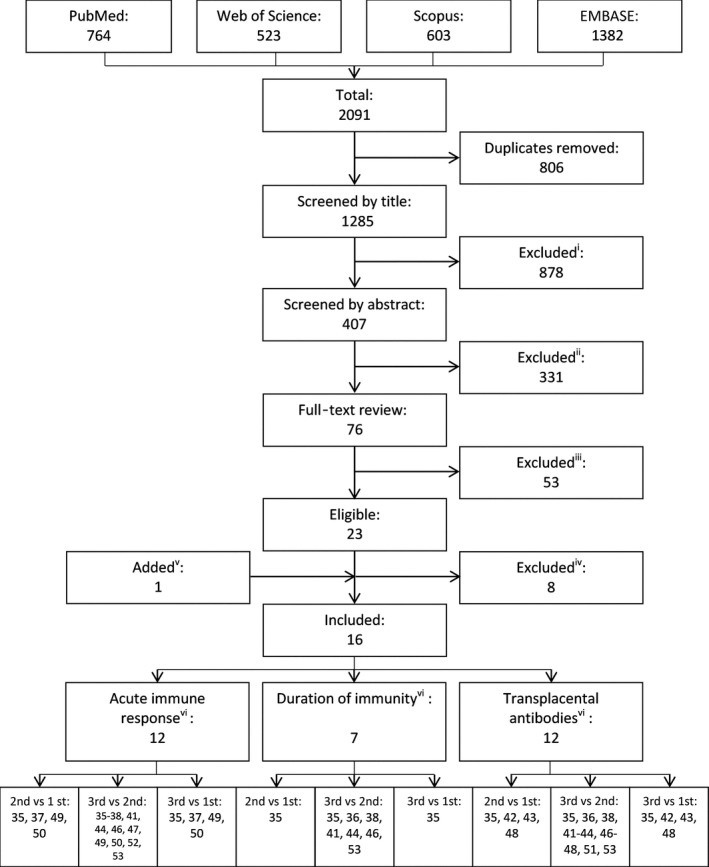
Flow diagram detailing the study inclusion/exclusion process. Reasons for inclusion/exclusion: (i) Article was a review, recommendation, statement, did not study pregnant women, did not report on immunogenicity, or did not study influenza vaccine. (ii) Article was a cost‐benefit analysis, studied vaccination uptake or attitudes, did not study pregnant women, did not report on immunogenicity, or did not study influenza vaccine. (iii) Article (n = 1) was a review of an included study, evaluated only adverse birth outcomes (n = 25), did not include any data on vaccination timing (n = 24), or only studied women in one trimester (n = 3). (iv) 19 studies contacted for stratified data: no response (n = 5), data not eligible due to time‐points measured (n = 1), declined (n = 1), data published in another included study (n = 1). (v) One article identified through hand‐searching of reference lists. (vi) References of studies included in each analysis

The records identified were assessed for eligibility in three phases by two independent reviewers (WC and RM) (Figure 1): screening by title, abstract and full‐text review. A third reviewer (NG) resolved any inconsistencies. Reference lists of papers that were identified through the database search were also searched for additional studies. If the study had not stratified GMTs by trimester of vaccination, authors were contacted to provide the required data. Grey literature was not searched.

### 2.3 Data extraction

Summary characteristics of each study (study period, design, sample size, location and population, trimester of vaccination and vaccine administered) and outcome measures (GMTs, standard deviation, confidence intervals, seroprotection and seroconversion rates) were extracted and tabulated by one reviewer (WC) (Table 1 and Table S2). The methodological validity and internal bias of all included studies were assessed using critical appraisal tools ROBINS‐I for non‐randomised trials (including single‐arm studies^35^, ^36^, ^37^, ^38^) and RoB 2.0 for randomised trials (Table S3).

**Table 1:** Summary characteristics of included studies

Study [ref] (year; setting)	Study design	Vaccine (season)^a^	Sample size with available data at each time‐point^b^											
			Pre‐vaccination			Post‐vaccination^c^			Mother at delivery			Cord blood^d^		
			1st TRI	2nd TRI	3rd TRI	1st TRI	2nd TRI	3rd TRI	1st TRI	2nd TRI	3rd TRI	1st TRI	2nd TRI	3rd TRI
Seasonal epidemics														
Kostinov et al^47^ (2015; MOW, RUS)	Prospective cohort	TIV Agrippal S1 (2 seasons: 2010/11, 2012/13)	–	27	21	–	27	21	–	–	–	–	23	19
Madhi et al^44^ (2014; SOW, RSA)	Randomised controlled trial	TIV Vaxigrip (2 seasons: 2011 & 2012)	–	59	83	–	59	83	–	59	83	–	35	58
Blanchard‐Rohner et al^52^ (2013; GVA, SUI)	Cross‐sectional	TIV Mutagrip (2010/11)	–	–	–	–	–	–	–	–	–	4	H1N1: 38; H3N2: 39; B: 38	H1N1: 56; H3N2: 57; B:56
Christian et al^48^ (2013; OH, USA)	Prospective cohort	TIV Fluarix (2011/12)	4	15	8	4	15	8	–	–	–	–	–	–
Garcia‐Putnam et al^46^ (2013; NC, USA)	Prospective cohort	TIV Fluarix (2011/12)	–	12	16	–	12	16	–	12	16	–	12	16
Lin et al^36^ (2013; TPE, TWN)	Prospective cohort	TIV AdimFlu‐S (2011/12)	–	16	30	–	16	27	–	15	29	–	15	27
Schlaudecker et al^49^ (2012; OH, USA)	Prospective cohort	TIV Fluarix (2011/12)	4	19	6	4	19	6	–	–	–	–	–	–
Eick et al^53^ (2011; USA)	Prospective cohort	TIVs (3 seasons: 2002/03, 2003/04, 2004/05)	–	–	–	–	–	–	–	–	–	–	H1N1: 123; H3N2: 60/60; B: 92/63	H1N1: 390; H3N2: 267/267; B:192/123
Yamaguchi et al^38^ (2009; TYO, JPN)	Prospective cohort	TIV FLUBIK HA (2007/08)	–	53	72	–	39	71	–	53	70	–	54	73
2009 pandemic														
Bischoff et al^50^ (2013; CPH, DEN)	Randomised controlled trial	U/A and MF59‐adj Focetria (A/H1N1/pdm09)	–	15 µg U/A: 41; 7.5 µg F/A: 31; 3.75 µg H/A: 11	15 µg U/A: 18; 7.5 µg F/A: 21; 3.75 µg H/A: 18	–	15 µg U/A: 41; 7.5 µg F/A: 31; 3.75 µg H/A: 11	15 µg U/A: 18; 7.5 µg F/A: 20; 3.75 µg H/A: 18	–	–	–	–	–	–
Chao et al^43^ (2013; TWN)	Prospective cohort	U/A and MF59‐adj AdimFlu‐S (A/H1N1/pdm09)	–	–	–	–	–	–	–	–	–	U/A: 8; Adj: 7	U/A: 15; Adj: 2	U/A: 8; Adj: 1
Fisher et al^42^ (2012; CO, USA)	Prospective cohort	Monovalent vaccine (A/H1N1/pdm09)	–	–	–	–	–	–	–	–	–	7	3	4
Horiya et al^35^ (2011; TYO, JPN)	Prospective cohort	Monovalent vaccine (A/H1N1/pdm09)	1D:17; 2D:17	1 D: 48; 2 D: 79	1 D: 35; 2 D: 29	1D: –; 2D:17/17	1 D: –; 2 D: 78/79	1 D: –; 2 D: 29/28	1 D: –; 2 D: 16	1 D: –; 2 D: 77	1 D: –; 2 D: 28	1D: 1; 2D: 16	1 D: 48; 2 D: 77	1 D: 35; 2 D: 28
Jackson et al^41^ (2011; SEA, USA)	Randomised controlled trial	Monovalent vaccine (A/H1N1/pdm09)	–	25 µg: 32; 49 µg: 42	25 µg: 23; 49 µg: 16	–	25 µg: 32/24; 49 µg: 42/37	25 µg: 23/14; 49 µg: 16/14	–	25 µg:26; 49 µg: 40	25 µg:15; 49 µg: 7	–	25 µg:25; 49 µg: 39	25 µg: 14; 49 µg: 7
Ohfuji et al^37^ (2011; OSA, JPN)	Prospective cohort	Monovalent vaccine (A/H1N1/pdm09)	26	46	77	26/26	46/46	77/77	–	–	–	–	–	–
Tsatsaris et al^51^ (2011; FRA)	Prospective cohort	Monovalent vaccine (A/H1N1/pdm09)	–	58	49	–	55	46	–	52	47	–	47	41

a TIV, trivalent influenza vaccine; Kostinov et al & Madhi et al used same vaccine in both seasons, respectively.

b TRI, trimester (1st TRI: <14 wk, 2nd TRI: ≥14 wk/<28 wk, 3rd TRI: ≥28 wk; where not otherwise specified, sample sizes are identical in analyses of each strain (H1N1, H3N2, B); Madhi et al participants were HIV‐negative; Eick et al display values for two H3N2 and two B strains, separated by a slash (2002/’03 & 2003/’04 vaccines contained the same strains, 2004/’05 contained different H3N2 & B strains); U/A: unadjuvanted, F/A: full‐adjuvanted, H/A: half‐adjuvanted; Adj: adjuvanted; D: dose; Horiya et al, Jackson et al and Ohfuji et al display post‐vaccination titres after 1st and 2nd dose, separated by a slash; “‐”: HI titres not measured at this time‐point.

c 1 month post‐vaccination: Kostinov et al, Madhi et al, Christian et al, Yamaguchi et al; 4 wk post‐vaccination: Lin et al, Garcia‐Putnam et al, Schlaudecker et al; 3 wk after vaccination: Bischoff et al, Horiya et al, Jackson et al, Ohfuji et al, Tsatsaris et al; 3 wk post‐2nd dose: Horiya et al & Jackson et al; 4 wk post‐2nd dose: Ohfuji et al

d Kostinov et al blood taken from newborn 2‐3 d post‐delivery, Madhi et al blood taken from newborn ≤ 7 d post‐delivery, Eick et al blood taken from newborn ≤ 14 d post‐delivery if cord blood was unavailable.

### 2.4 Analysis

Three analyses of study data were conducted. First, the effect of vaccination trimester on acute immune response was measured by calculating a ratio of GMT fold increases (GMFI) comparing women vaccinated in different trimesters; this ratio used a pre‐vaccination GMT and a post‐vaccination GMT (either three weeks, four weeks, or one month after vaccination). Second, the effect of vaccination trimester on antibody persistence was measured by calculating a ratio of GMT fold decreases (GMFD) comparing women vaccinated in different trimesters; this ratio used a post‐vaccination GMT (measured either three weeks, four weeks, or one month after vaccination) and a maternal GMT measured at the time of delivery. Third, the effect of vaccination trimester on transplacental antibodies was measured by calculating a ratio of cord blood GMTs (GMR) comparing women vaccinated in different trimesters; this ratio used only the cord blood time‐point.

Ratios of GMTs were used to account for anticipated differences in baseline seropositivity between seasons. Relative change in GMT is an appropriate measure to account for this heterogeneity, allowing the comparison of women vaccinated with different vaccines, undergoing different protocols and with different demographic characteristics.

Where not otherwise reported, standard errors for GMTs at each time‐point were calculated from reported 95% confidence intervals (CI) using the t‐distribution or from standard deviations. Standard errors of ratios were calculated using the z‐distribution or, if the sample size did not change between time‐points, the t‐distribution. All analyses were conducted on the logarithmic scale and back‐transformed to the original scale. Heterogeneity was examined using forest plots and the I^2^ statistic. Due to the small number of studies included, funnel plots to check for publication bias were not produced.^39^ Data were summarised using a random‐effect (DerSimonian and Laird) model to accommodate between‐study heterogeneity in true effects, and the estimate and 95% CI of the effect were presented on the logarithmic scale.^40^

**Figure 2:**
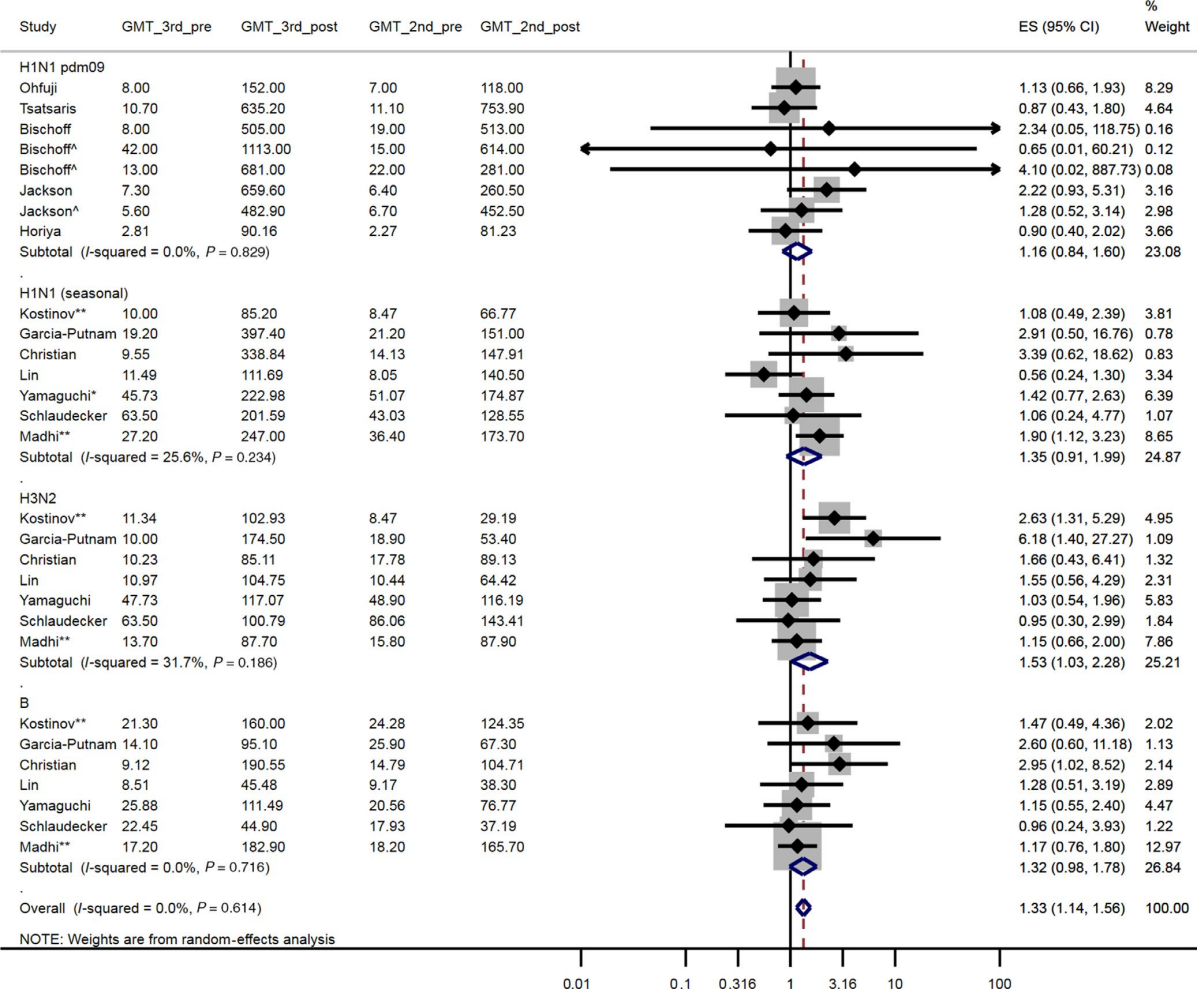
A forest plot of the geometric mean fold increase (GMFI) ratio, pre‐vaccination to post‐vaccination, comparing women vaccinated in the 3rd trimester with women vaccinated in the 2nd trimester. ^ Bischoff et al 7.5 µg & 3.75 µg groups, respectively (as opposed to the 15 µg group); Jackson et al 49 µg group (as opposed to the 25 µg group). * Yamaguchi et al studied a non‐pdm09 H1N1 strain (2007/08 season). ** Kostinov et al & Madhi et al was conducted over two influenza seasons (using same vaccine in both seasons). GMT_3^rd^_pre: geometric mean titre (GMT) pre‐vaccination, 3^rd^ trimester vaccination. GMT_3^rd^_post: GMT post‐vaccination, 3^rd^ trimester vaccination. GMT_2^nd^_pre: GMT pre‐vaccination, 2^nd^ trimester vaccination. GMT_2^nd^_post: GMT post‐vaccination, 2^nd^ trimester vaccination. ES (95% CI): Effect size (GMFI) (95% confidence interval)

**Figure 3:**
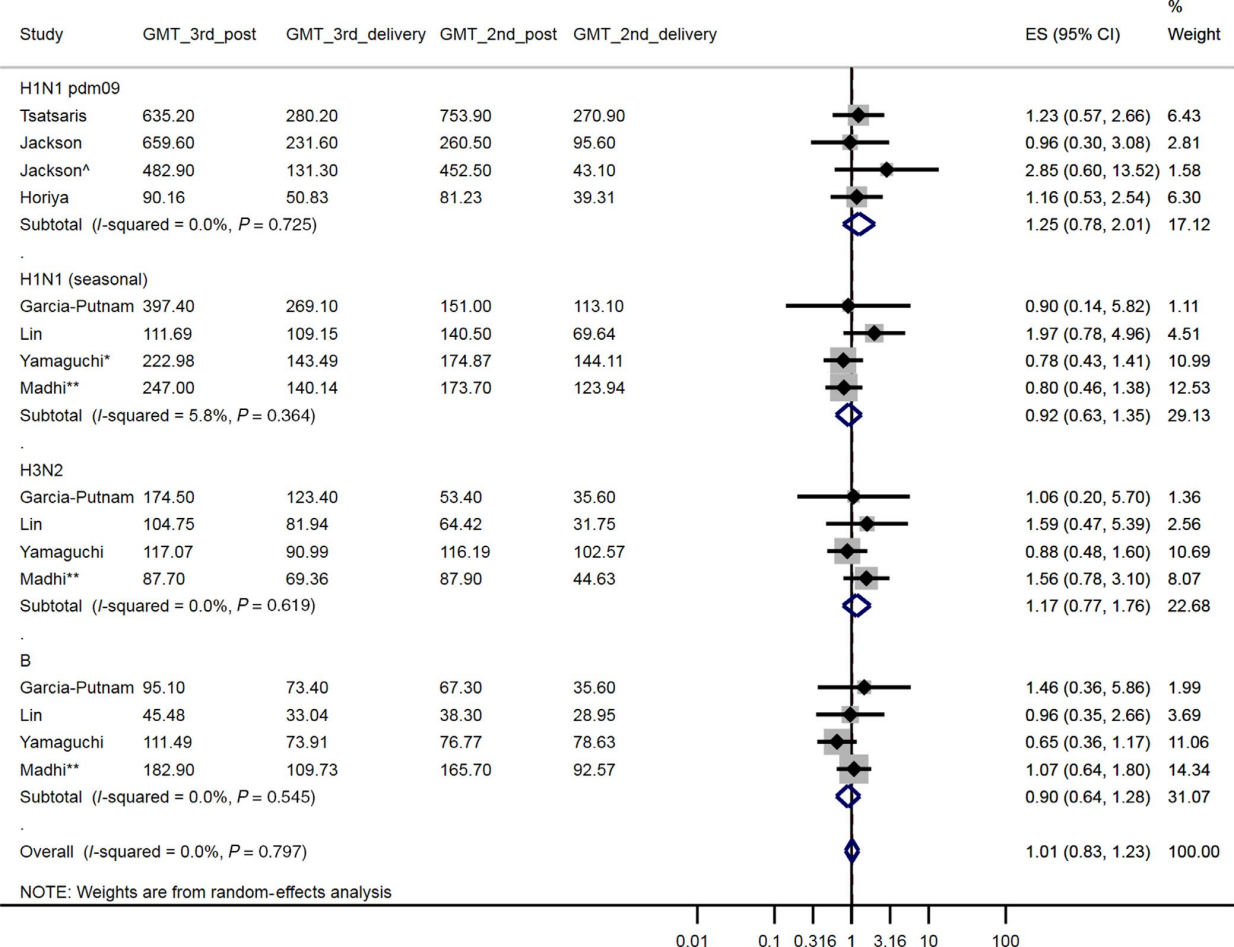
A forest plot of the geometric mean fold decrease (GMFD) ratio, post‐immunisation to delivery, comparing women vaccinated in the 3^rd^ trimester with women vaccinated in the 2^nd^ trimester. ^ Jackson et al 49 µg group (as opposed to the 25 µg group). * Yamaguchi et al studied a non‐pdm09 H1N1 strain (2007/08 season). ** Madhi et al was conducted over two influenza seasons (using same vaccine in both seasons). GMT_3^rd^_ post: geometric mean titre (GMT) post‐vaccination, 3^rd^ trimester vaccination. GMT_3^rd^_delivery: GMT in mother at delivery, 3^rd^ trimester vaccination. GMT_2^nd^_ post: GMT post‐vaccination, 2^nd^ trimester vaccination. GMT_2^nd^_delivery: GMT in mother at delivery, 2^nd^ trimester vaccination. ES (95% CI): Effect size (GMFD) (95% confidence interval)

Timing of vaccination was stratified by trimester (1st trimester: <14 weeks, 2nd trimester: 14‐27 weeks, 3rd trimester: ≥28 weeks). Accordingly, three comparisons were made: 2nd‐trimester vaccination compared with 1st‐trimester vaccination, the 3rd trimester compared with the 2nd and the 3rd trimester compared with the 1st. For studies that included a two‐dose vaccine group, these data were analysed in separate meta‐analyses (trimester of vaccination defined by the timing of the first dose).^35^, ^37^, ^41^ Seven primary meta‐analyses (Figure 2, 3, 4 & Figures S1, S7, S12, S16) were conducted, plus four separate meta‐analyses of results after a second dose (Figures S2, S3, S8, S9). Within each meta‐analysis, results were presented across all virus strains and by virus strain. We did not adjust for multiple testing.

**Figure 4:**
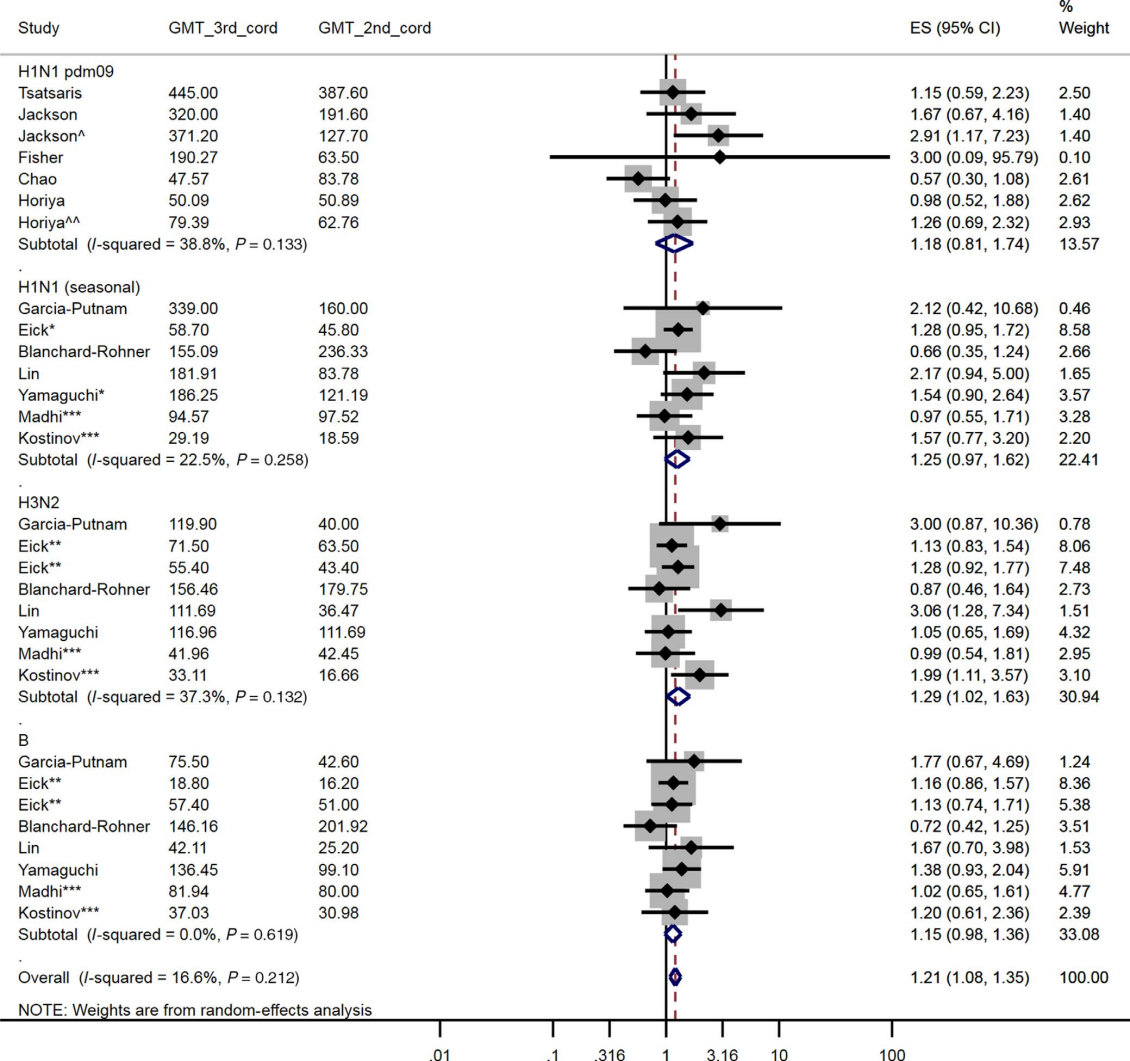
A forest plot of the cord‐blood GMT ratio comparing women vaccinated in the 3^rd^ trimester with women vaccinated in the 2^nd^ trimester. ^ Jackson et al 45 µg dose group (as opposed to the 25 µg dose group) – all women had received two vaccine doses. ^^ Horiya et al two vaccine dose group (as opposed to the one vaccine dose group). * Eick et al & Yamaguchi et al studied non‐pdm09 H1N1 strain (2002‐05 seasons and 2007/08 season, respectively). ** Eick et al contained different H3N2 and two B strains in two of the three seasons (‘02/’03 & ‘03/’04 vaccines contained the same strains, ‘04/’05 contained different H3N2 & B strains). *** Madhi et al and Kostinov et al were conducted over two influenza seasons (using same vaccine in both seasons). GMT_3^rd^_cord: geometric mean titre (GMT) in cord‐blood at delivery, 3^rd^ trimester vaccination. GMT_2^nd^_cord: GMT in cord‐blood at delivery, 2^nd^ trimester vaccination. ES (95% CI): Effect size (GMR) (95% confidence interval)

We also examined seroprotection and seroconversion rates reported in the included studies (Table S2). Where possible, we explored these outcomes stratified by trimester of vaccination; however, a limited number of studies reported these stratified data. We included some statistical results from the original papers (eg p‐values from comparisons of seroprotection and seroconversion proportions).

For the acute immune response and transplacental antibody outcomes, we conducted three sensitivity analyses for the 3rd‐ versus 2nd‐trimester comparison in which we restricted the studies included in the meta‐analysis to those that also vaccinated women in the 1st trimester (Figures S4, S5, S15). This sensitivity analysis allows us to explore whether the findings based on studies that included women vaccinated in the 1st trimester (as well as the 2nd and 3rd) may have differed from those of studies that included only women vaccinated in the 2nd and 3rd trimesters due to participant recruitment. This analysis was not performed for the antibody persistence outcome because there was only one study that included women vaccinated in the 1st trimester.^35^ Six other sensitivity analyses were undertaken whereby studies with concerns of bias (due to small sample size^42^, ^43^ or unbalanced loss to follow‐up between study groups^38^, ^41^, ^44^) were excluded (Table S3).

All analyses included all available cases with the unit of analysis being the participant. Data preparation and manipulation were undertaken using Microsoft Excel, and all meta‐analyses and forest plots were produced in Stata 14.2.^45^

## 3 RESULTS

### 3.1 Literature search

After duplicates were removed, 1,285 articles were identified from the electronic databases search (Figure 1). 878 and 331 articles were excluded after screening by title and abstract, respectively. Full‐text assessment of the remaining 76 studies left 23 eligible studies. One additional study was identified from the reference lists of the included studies. Of these 24 studies, five had complete data. We contacted authors of the other 19 studies, 11 of whom provided additional data.

In total, 16 studies were included. Nine were conducted during seasonal epidemics (two prior to the 2009 pandemic) and seven during the H1N1/pdm09 year; 12 were cohort studies, three were randomised controlled trials (RCTs), and one was a cross‐sectional study (Table 1). One was a conference abstract.^46^ Spanning a decade (2002‐2012), this systematic review includes studies conducted in eight different countries and during eight different influenza seasons.

### 3.2 Acute immune response

Twelve studies measured both baseline HI titres immediately prior to vaccination and post‐vaccination HI titres (Table S2). Post‐vaccination blood draw occurred at 1 month after vaccination in four studies,^38^, ^44^, ^47^, ^48^ at 4 weeks in three studies^36^, ^46^, ^49^ and at 3 weeks in five studies.^35^, ^37^, ^41^, ^50^, ^51^ Horiya et al^35^ and Jackson et al^41^ also measured HI titres 3 weeks after a second dose, whereas Ohfuji et al^37^ measured titres at 4 weeks after the second dose (compared to 3 weeks after the first dose). These pre‐ and post‐vaccination time‐points allowed us to assess the production of antibodies induced by vaccination (geometric mean titre fold increase (GMFI)). For this outcome, four studies^35^, ^37^, ^48^, ^49^ included women vaccinated in any trimester while the remaining eight studies included only women vaccinated in the 2nd and 3rd trimester.

#### 3.2.1 2nd versus 1st trimester

Across all influenza strains, the GMFI for women vaccinated in the 2nd trimester was 1.45 [95% CI: 0.98, 2.14] times greater than those vaccinated in the 1st trimester (Figure S1; 4 studies, 210 participants). Subgroup analyses within each influenza subtype also revealed effect sizes in this direction. Only Horiya et al^35^ had a lower GMFI in the 2nd trimester (after 1st dose: 0.87 [0.33, 2.26]).

After a second dose, Horiya et al^35^ and Ohfuji et al^37^ both showed a greater GMFI for women vaccinated in the 2nd trimester than 1st trimester (1.16 [0.44, 3.08] and 1.16 [0.59, 2.29], respectively) (Figure S2; 2 studies, 168 participants).

#### 3.2.2 3rd versus 2nd trimester

The GMFI for women vaccinated in the 3rd trimester was 1.33 [95% CI: 1.14, 1.56] times greater than those vaccinated in the 2nd trimester (Figure 2; 12 studies, 1028 participants). Studies by Kostinov et al^47^ and Madhi et al^44^ were conducted over two influenza seasons and both had robust results for at least one subtype (H3N2 for Kostinov et al^47^:2.63 [1.31, 5.29] and H1N1 for Madhi et al^44^:1.90 [1.12, 3.23]).

After a second dose, the GMFI for women vaccinated in the 3rd trimester was 1.34 [0.93, 1.93] times greater than for women vaccinated in the 2nd trimester (Figure S3; 3 studies, 344 participants). However, this trend was not consistent across all three studies.

Restricting the meta‐analysis to only studies that also vaccinated women in the 1st trimester^35^, ^37^, ^48^, ^49^ slightly lowered the pooled GMFI from 1.33 [1.14, 1.56] to 1.25 [0.89, 1.76] (Figures S4 and S5; 4 studies/279 participants and 2 studies/231 participants, respectively). In another sensitivity analysis, excluding Yamaguchi et al^38^ due to risk of internal bias, the pooled GMFI was 1.36 [1.15, 1.61] (Figure S6; 11 studies, 903 participants).

#### 3.2.3 3rd versus 1st trimester

The clarity of a dose‐response relationship by trimester is strengthened by comparing women vaccinated in the 3rd and 1st trimester: the GMFI for women vaccinated in the 3rd trimester was 1.96 [95% CI: 1.01, 3.82] times greater than those vaccinated in the 1st trimester (Figure S7; 4 studies, 171 participants). There was also a similar trend after a second dose (1.56 [0.89, 2.74]; Figure S8; 2 studies, 149 participants).

### 3.3 Antibody persistence

In addition to the post‐vaccination blood draw, seven studies^35^, ^36^, ^38^, ^41^, ^44^, ^46^, ^51^ also measured HI titres in the mother at delivery (Table S2). Thus, we can quantify the reduction in antibodies between the post‐vaccination immune response and delivery (geometric mean titre fold decrease (GMFD)). All seven studies included women vaccinated in the 2nd and 3rd trimester, while only one^35^ included women in all three trimesters.

#### 3.3.1 2nd versus 1st trimester

Horiya et al^35^ were the only study to include women vaccinated in all three trimesters; therefore, no meta‐analysis was possible. In this study, the fold reduction in GMT was greater for women vaccinated in the 1st trimester compared with women vaccinated in the 2nd trimester (1.59 [95% CI: 0.56, 4.54]). This difference was smaller when comparing women who received two vaccine doses during their pregnancy (1.19 [0.41, 3.49]).

#### 3.3.2 3rd versus 2nd trimester

The fold reduction in GMT from immunisation to delivery was similar for women vaccinated in the 2nd and 3rd trimesters (1.01 [95% CI: 0.83, 1.23]) (Figure 3; 7 studies, 658 participants). A notable exception was the study by Yamaguchi et al,^38^ in which the GMFD was smaller following 2nd trimester immunisation across all strains (H1N1: 0.78 [0.43, 1.41], H3N2: 0.88 [0.48, 1.60], B: 0.65 [0.36, 1.17]).

After a second dose, the GMFD between immunisation and delivery for women vaccinated in the 3rd trimester was less than for women vaccinated in the 2nd trimester (1.28 [0.70, 2.35] times; Figure S9; 2 studies, 202 participants).

A sensitivity analysis in which Madhi et al^44^ and the 49 µg dose group of Jackson et al^41^ were excluded due to risk of internal bias (Table S3) had a minor impact on the effect size (after 1 dose: 0.97 [0.76, 1.24]; after 2 doses: 1.10 [0.57, 2.10]; Figures S10 and S11; 7 studies/600 participants and 2 studies/148 participants, respectively).

#### 3.3.3 3rd versus 1st trimester

Comparable to the 2nd‐ versus 1st‐trimester analysis of the same study, Horiya et al^35^ demonstrated that the fold reduction in GMT was greater for women vaccinated in the 1st trimester compared with women vaccinated in the 3rd trimester (1.86 [95% CI: 0.60, 5.76]). This difference was smaller when comparing women who received two vaccine doses during their pregnancy (1.28 [0.40, 4.05]).

### 3.4 Transplacental antibodies

Twelve studies measured HI titres in cord blood at delivery (Table S2). Four studies^35^, ^42^, ^43^, ^52^ included women vaccinated in any trimester while eight studies included only women vaccinated in the 2nd and 3rd trimester. This time‐point enables the direct comparison of antibodies transferred to the foetus by trimester of vaccination (geometric mean titre ratio (GMR)).

#### 3.4.1 2nd versus 1st trimester

Women vaccinated in the 2nd trimester had a higher cord blood GMT (1.64 [95% CI: 1.21, 2.24]) than those immunised earlier in pregnancy (Figure S12; 4 studies, 178 participants). All point estimates were greater than one, and the pooled effect size in the subgroup analysis of the H1N1pdm09 strain was robust (1.50 [1.03, 2.19]). All women in Horiya et al^35^ received two vaccine doses, and the cord blood GMR by trimester in this study was similar to other studies that administered only one dose (1.59 [0.75, 3.38]). In Chao et al,^43^ the cord blood GMTs post‐1st and post‐2nd trimester vaccination with adjuvanted vaccine were less different than after vaccination with unadjuvanted vaccine (unadjuvanted GMR: 1.62 [0.89, 2.94]; adjuvanted GMR: 1.22 [0.62, 2.39]) although confidence intervals of both ratios included 1.

A sensitivity analysis in which results from Fisher et al^42^ and the adjuvanted vaccine group in Chao et al^43^ were excluded due to risk of internal bias (Table S3) had minimal impact on the pooled effect size (1.76 [1.23, 2.51], Figure S13; 3 studies, 159 participants).

#### 3.4.2 3rd versus 2nd trimester

Consistent with the 2nd‐ versus 1st‐trimester comparison above, women vaccinated in the 3rd trimester had a 1.21 [95% CI: 1.08, 1.35] times higher cord blood GMT than women vaccinated in the 2nd trimester (Figure 4; 12 studies, 1332 participants). The results from Horiya et al^35^ and Jackson et al^41^ suggest that this relationship between vaccination in the later trimester and higher cord blood titres is accentuated with a second dose (3 weeks after the first) or with a higher dose. Eick et al^53^ and Yamaguchi et al^38^ studied different seasonal H1N1 strains prior to 2009; however, their GMT cord blood ratios by trimester are similar to studies of H1N1pdm09 vaccines. Furthermore, Eick et al^53^ took place over three influenza seasons with three vaccines comprising two unique H3N2 and B strains (the H1N1 strain was the same in all three vaccines); the cord blood GMTs for both H3N2 and B strains between women vaccinated in the 3rd and 2nd trimesters were virtually equivalent.

A sensitivity analysis in which results from Fisher et al^42^ and the 45 µg dose group in Jackson et al^41^ were excluded due to risk of internal bias (Table S3) had minimal impact on the pooled effect size (1.19 [1.07, 1.33], Figure S14; 11 studies, 1279 participants).

Restricting the meta‐analysis to only studies that also vaccinated women in the 1st trimester^35^, ^42^, ^43^, ^52^ changed the direction of the pooled effect (0.82 [0.64, 1.06], (Figure S15; 4 studies, 314 participants)). However, this result is strongly influenced by a reduction in studies with GMT cord blood ratios greater than one.

#### 3.4.3 3rd versus 1st trimester

Women vaccinated in the 3rd trimester had a cord blood GMT 1.44 [95% CI: 0.95, 2.19] times higher than those immunised in the 1st trimester (Figure S16; 4 studies, 131 participants). Even though Horiya et al^35^ include only women who received two vaccine doses, the cord blood GMR by trimester in this study was similar to other single‐dose studies (2.01 [0.84, 4.82]). Fisher et al^42^ had a much larger effect size but were also far less precise (8.62 [0.38, 194.16]).

A sensitivity analysis in which Fisher et al^42^ were excluded due to risk of internal bias (Table S3) had minimal impact on the pooled effect size (1.40 [0.93, 2.10], Figure S17; 3 studies, 121 participants).

### 3.5 Seroprotection and seroconversion

#### 3.5.1 Acute immune response

With the exception of women vaccinated in the 2nd trimester in Kostinov et al,^47^ seroprotection and seroconversion rates were consistently in excess of 75% irrespective of the timing of vaccination. Compared with women vaccinated in the 2nd trimester, Kostinov et al^47^ reported that women vaccinated in the 3rd trimester had a significantly higher seroconversion rate (*P* < 0.01) against the H3N2 subtype (3rd trimester: 73%; 2nd trimester: 30%) and influenza B (3rd trimester: 82%; 2nd trimester: 52%). Ohfuji et al^37^ only found a significant difference in seroconversion rates by trimester four weeks after administering a second vaccine dose (1st trimester: 76%, 2nd trimester: 91%, 3rd trimester: 94% (*P* = 0.02)). Similarly, Horiya et al^35^ found a non‐significant increase in HI titres after a second dose; there were no differences by trimester of vaccination (90% seroconversion rate regardless of timing). In addition, neither Tsatsaris et al^51^ nor Garcia‐Putnam et al^46^ found any significant differences in seroconversion or seroprotection rates by trimester, although Garcia‐Putnam et al^46^ reported a similar trend with a seroprotection rate of 100% against the H1N1 subtype for women vaccinated in the 3rd trimester and 75% for those vaccinated in the 2nd trimester.

The remaining studies did not stratify seroprotection and seroconversion rates by trimester at this time‐point^36^, ^38^, ^41^, ^48^, ^49^, ^50^; generally, seroprotection rates were very high (H1N1: 88‐100%; H3N2: 81‐100%; B: 57‐83%), while seroconversion rates were low (H1N1: 51‐97%; H3N2: 10‐63%; B: 21‐63%) (see Limitations).

#### 3.5.2 Antibody persistence

Compared with women vaccinated in the 2nd trimester, Kostinov et al^47^ demonstrated a greater seroprotection rate for 3rd trimester vaccinated women in the days following delivery (*P* < 0.01). Furthermore, comparing HI titres one month after vaccination and at delivery, Yamaguchi et al^38^ found women vaccinated in the 3rd trimester maintained elevated antibody levels (>1:40) more effectively than did women vaccinated in the 2nd trimester (2nd trimester: 66% ± 27% (standard deviation); 3rd trimester: 94% ± 31% (*P* < 0.001)). In contrast, Tsatsaris et al^51^ reported similar seroprotection rates at delivery for both women vaccinated in the 2nd and 3rd trimester (2nd trimester: 92% [95% CI: 81%, 98%]; 3rd trimester: 91% [80%, 98%]). Protection extending beyond delivery was also measured by Kostinov et al,^47^ who found no difference in seroprotection rates for women vaccinated in different trimesters at three or six months after delivery; however, both groups had experienced a significant decrease in seroprotection.

Further evidence of waning protection was reported by Lin et al^36^ and Fisher et al,^42^ who both found a significant decrease in antibody titres between vaccination and delivery, with Lin et al^36^ using linear regression to estimate that HI titres fall below 1:40 after 150 days. Horiya et al^35^ reported a non‐significant association between higher HI titres at delivery and vaccination in later stages of pregnancy, and for women who received two vaccine doses. Jackson et al^41^ also established that a longer vaccination‐to‐delivery interval resulted in lower HI titres at delivery (*P* < 0.05), however did not find a difference in seroprotection rates in the mother at delivery between 25 µg and 49 µg dose groups (25 µg: 85% [71%, 94%]; 49 µg: 62% [46%, 75%]). Finally, all three vaccine groups in Bischoff et al^50^ maintained high seroprotection and seroconversion rates for at least three months after vaccination, and only the 7.5 µg full‐adjuvanted group showed a significant decrease in protection after 10 months (3 months: seroconversion rate: 95% [82%, 99%], seroprotection rate: 96% [88%, 100%]; 10 months: seroconversion rate: 59% [39%, 76%], seroprotection rate: 70% [55%, 83%]).

Even though there was some evidence of higher antibody levels at delivery in women vaccinated later in pregnancy, seroprotection and seroconversion rates at delivery were generally high regardless of vaccination timing.^36^, ^41^, ^42^, ^43^, ^46^

#### 3.5.3 Transplacental antibodies

Kostinov et al^47^ found that infants born to women vaccinated in the 3rd trimester had significantly higher seroprotection rates 2‐3 days after delivery (*P* < 0.01), compared with infants born to women vaccinated in the 2nd trimester (2nd trimester: H1N1: 39%, H3N2: 37%; 3rd trimester: H1N1: 68%, H3N2: 76%). Both groups of infants had decreased seroprotection rates three months later; however, rates remained higher (*P* < 0.01) for the 3rd trimester group (2nd trimester: H1N1: 16%, H3N2: 11%, B: 26%; 3rd trimester: H1N1: 26%, H3N2: 37%, B: 42%). In contrast, neither Tsatsaris et al^51^ nor Eick et al^53^ found any significant difference in cord blood seroprotection rates between women vaccinated in the 2nd or 3rd trimesters. Furthermore, Yamaguchi et al^38^ reported that the ratio between HI titre in maternal blood at delivery and cord blood was higher for women vaccinated in the 2nd trimester (2nd trimester: 161%; 3rd trimester: 127% (*P* = 0.02)). Finally, compared with no vaccination, Blanchard‐Rohner et al^52^ found vaccination less than 15 days before delivery did not result in a significant increase in seroprotection rate at delivery.

The remaining studies did not stratify seroprotection and seroconversion rates by trimester at this time‐point.^35^, ^36^, ^41^, ^43^, ^44^ While Jackson et al^41^ found no difference in cord blood seroprotection between 25 µg and 49 µg dose groups, Horiya et al^35^ found non‐significantly higher rates for women vaccinated in later stages of pregnancy and for women who received two vaccine doses.

### 3.6 Risk of bias

Largely influenced by the nature of the intervention and the outcome measures, we determined that there was low risk of internal methodological bias in all included studies (Table S3). However, the main limitations were low numbers of participants, missing data or loss to follow‐up (Table 1). In Jackson et al,^41^ 56% of participants in the 49 µg dose group were missing data at delivery, while in Madhi et al,^44^ 36% of women vaccinated in the 2nd trimester were missing data at delivery (using per‐protocol participants). Similarly, 26% of those vaccinated in the 2nd trimester in Yamaguchi et al^38^ were missing data at 28 days post‐vaccination. Those vaccinated with an adjuvanted vaccine in Chao et al^43^ (ten in total) included only two participants in the 2nd trimester and only one in the third trimester (not included in the analysis). Fisher et al^42^ also had a very small sample size (1st trimester: 7, 2nd trimester: 3, 3rd trimester: 4). The effects of these studies on the pooled effect sizes were explored through sensitivity analyses (Figures S6, S10, S11, S13, S14, S17).

## 4 DISCUSSION

### 4.1 Summary of findings

This systematic review explored the association between the timing of an influenza vaccination given during pregnancy and immunogenicity in the mother and newborn. The three main findings of this review were as follows. First, women vaccinated later during pregnancy had a greater immune response to vaccination. This effect size increased from the 1st to 3rd trimester. Second, maternal antibodies at delivery were reduced by a similar factor relative to post‐immunisation titres, regardless of whether women were vaccinated in the 2nd or 3rd trimester. This observation suggests that antibodies wane faster in women vaccinated later in pregnancy; however, this hypothesis could not be explored further due to the low number of studies that vaccinated women in the 1st trimester. Regardless, antibody waning seems to occur over a fairly short time frame; some studies have estimated the antibody half‐life to be as short as seven weeks.^54^, ^55^ Third, despite the observation that GMT at delivery was consistent across trimesters, there was strong evidence that vaccination in a later trimester increased the transfer of antibodies to the foetus.

To gain further insight into the impact of vaccination timing, we reported on the public health–relevant outcomes of seroprotection and seroconversion. An HI titre of 1:40 is recognised as an immunologic correlate corresponding to a 50% reduction in the risk of contracting influenza.^56^ Even though some of the studies included in this review reported significantly higher seroprotection or seroconversion rates for women vaccinated in later trimesters at both the acute post‐vaccination time‐point and at delivery in the mother and cord blood, most women still achieved sufficient protection regardless of vaccination timing. Other studies not meeting this review's inclusion criteria have found no difference in seroprotection rates by vaccination trimester.^12^, ^57^, ^58^, ^59^

Some study designs included administering two vaccine doses, higher antigen doses or adjuvanted vaccines.^35^, ^37^, ^41^, ^43^, ^50^ By trimester, the immune response was not greatly impacted by higher doses or adjuvanted vaccines; neither was a substantial increase in GMT conferred after a second dose. However, those who received two doses or a higher dose tended to have higher levels of antibodies at delivery and also transferred more antibodies to the foetus.

### 4.2 Limitations

Of the original publications, only six reported the effect of vaccination timing on vaccine immunogenicity in detail with stratification by trimester. Thus, much of the data acquired for this review were not originally collected to address our objectives. Observational studies are also prone to inherent biases, and many of the included studies only had small numbers of participants. While RCTs are desirable for addressing the impacts of antenatal vaccination timing on vaccine immunogenicity, there are limitations on study design due to the ethical issues raised by delaying vaccination. Comparing the usefulness of multiple doses is a potential alternative. Furthermore, some of the included studies used vaccines not licensed for pregnant women, potentially limiting the generalisability of their results.

All studies in this review included women vaccinated in the second and third trimesters, but only seven studies included women vaccinated in the first trimester (likely influenced by the short time between pregnancy diagnosis and the end of first trimester). This limited the comparisons of vaccine immunogenicity against women vaccinated in the first trimester. The robustness of our results is likely affected by the low number of studies available for inclusion in these meta‐analyses (Figure 1). However, the I^2^ values were less than 44% in all our meta‐analyses, indicating low between‐study heterogeneity. Finally, we interpreted our results by considering overall trends rather than statistical significance and did not account for multiple comparisons.

Immunity prior to vaccination can both affect the immune response generated from the vaccine and mask the true immunogenicity of the vaccine. Seroprotection rates may over‐estimate immunogenicity, while seroconversion rates may result in under‐estimation if there is high baseline population protection. We used fold increases and fold decreases in GMT in an attempt to control for the pre‐vaccination immune state—a valid method when combined into a meta‐analysis.^60^ Furthermore, exposure to wild‐type influenza virus between vaccination and delivery may have impacted HI titres measured at delivery.

Finally, the widely accepted standard correlate of protection (ie based on challenge studies in healthy adults, that an HI titre >1:40 corresponds to a 50% reduction in the risk of contracting influenza) is not grounded in strong evidence.^61^, ^62^ While a higher HI titre is predictive of some protection, there is evidence to suggest that neuraminidase inhibition (NAI) titre is more predictive of protection and reduced infection.^63^ NAI titres were not measured in any of the included studies. There remains a need to standardise serological assays and better define correlates of protection, which may vary according to individual characteristics, populations, age groups and vaccine types.

## 5 IMPLICATIONS AND CONCLUSION

Vaccinating a woman in early pregnancy will provide protection against influenza for a greater proportion of pregnancy, but may increase the probability that this immunity will not last until delivery. Lower antibody levels at delivery may reduce transplacental antibodies and the benefit to the newborn, and loss of immunity in the mother may increase the chance of her becoming a viral source to the newborn. A similar issue arises with antenatal pertussis vaccination, where there is recent evidence that immunogenicity is higher in the second trimester compared with the third trimester, which has resulted in some countries bringing forward their recommendations for the optimal timing of pertussis immunisation in pregnancy.^64^ Furthermore, for women vaccinated early during pregnancy, but late in the influenza season, clinical protection may be reduced if the circulating strain of the following influenza season does not match the strain included in the previous season's vaccine, regardless of whether antibody levels remain high. Given that women immunised earlier in pregnancy show evidence of immune waning by the point of delivery, our findings support current recommendations for women immunised early in their pregnancy to receive a second dose if they are still pregnant in the following influenza season.

Due to the increased risk of adverse birth outcomes caused by maternal influenza infection, and the subsequent increased risk of lifelong chronic diseases associated with these birth outcomes, the economic burden of maternal infection is substantial.^65^ There is recent evidence that the risk of foetal death and other adverse birth outcomes is highest for women who become infected with seasonal influenza during their first trimester.^66^ That research compounds the complexity of optimising the scheduling of influenza vaccination for pregnant women and, in conjunction with our findings, highlights that the infant is at risk both in utero and after birth. The protection of the infant from adverse birth outcomes and infection in early life has not been measurable outcomes in this review. Nevertheless, this is a key aspect of antenatal vaccination policy and there is an extensive amount of documented interest in these benefits.^51^, ^52^, ^67^, ^68^, ^69^

While studies of vaccine effectiveness and efficacy were not included in this systematic review, the increased immune response by gestational age suggests that post‐vaccination influenza infection might be less likely for women vaccinated in later trimesters. However, preventing clinical disease depends more on the timing of vaccination relative to the seasonal influenza epidemic rather than to gestational age. For example, vaccination should not be delayed for a woman in her first trimester if the influenza season has begun and the vaccine is available.

The findings of this systematic review are informative and relevant to current vaccine scheduling for pregnant women. We found that women vaccinated later in pregnancy had a stronger immune response and transferred more antibodies to the foetus.

A limited number of published studies have considered the impact of vaccine timing on maternal and infant protection. We need to understand the full implications of vaccination timing for protection of mother, foetus and newborn. This knowledge is key to enabling future health policies to optimise protection for both mother and infant, developing future vaccine scheduling recommendations, and informing professional advice. In addition, a better understanding of the benefits of influenza vaccination during pregnancy may help increase vaccination rates among pregnant women.

## AUTHOR CONTRIBUTIONS

WC, NG, JF, SB, JMcV and RM designed the study, conducted the analysis and prepared the manuscript for publication. The following co‐authors provided original data and approved the final manuscript for submission: SAM, MCN, LMC, SYL, CNL, KY, HB, BC, ASC, GBR, ES and BF.
