# Supplementary material for: Optimal timing of influenza vaccine during pregnancy: A systematic review and meta‐analysis
Source: Influenza Other Respir Viruses. 2019 Jun 5;13(5):438–52. doi: 10.1111/irv.12649 (PMC6692549; doi:10.1111/irv.12649)
Supplement: Supplementary file 1 [file IRV-13-438-s001.docx]

***Supplementary Table 1****. PRISMA checklist*

| **Section/topic** | **#** | **Checklist item** | **Reported on page/Table/Figure** |
| --- | --- | --- | --- |
| **TITLE** | | |  |
| Title | 1 | Identify the report as a systematic review, meta-analysis, or both. | 1 |
| **ABSTRACT** | | |  |
| Structured summary | 2 | Provide a structured summary including, as applicable: background; objectives; data sources; study eligibility criteria, participants, and interventions; study appraisal and synthesis methods; results; limitations; conclusions and implications of key findings; systematic review registration number. | 4 |
| **INTRODUCTION** | | |  |
| Rationale | 3 | Describe the rationale for the review in the context of what is already known. | 5-7 |
| **METHODS** | | |  |
| Objectives | 4 | Provide an explicit statement of questions being addressed with reference to participants, interventions, comparisons, outcomes, and study design (PICOS). | 7 |
| Protocol and registration | 5 | Indicate if a review protocol exists, if and where it can be accessed (e.g., Web address), and, if available, provide registration information including registration number. | NA |
| Eligibility criteria | 6 | Specify study characteristics (e.g., PICOS, length of follow-up) and report characteristics (e.g., years considered, language, publication status) used as criteria for eligibility, giving rationale. | 7, 8 |
| Information sources | 7 | Describe all information sources (e.g., databases with dates of coverage, contact with study authors to identify additional studies) in the search and date last searched. | 8 |
| Search | 8 | Present full electronic search strategy for at least one database, including any limits used, such that it could be repeated. | 8 |
| Study selection | 9 | State the process for selecting studies (i.e., screening, eligibility, included in systematic review, and, if applicable, included in the meta-analysis). | 7, 8, Figure 1 |
| Data collection process | 10 | Describe method of data extraction from reports (e.g., piloted forms, independently, in duplicate) and any processes for obtaining and confirming data from investigators. | 8 |
| Data items | 11 | List and define all variables for which data were sought (e.g., PICOS, funding sources) and any assumptions and simplifications made. | 8 |
| Risk of bias in individual studies | 12 | Describe methods used for assessing risk of bias of individual studies (including specification of whether this was done at the study or outcome level), and how this information is to be used in any data synthesis. | 9, 11 |
| Summary measures | 13 | State the principal summary measures (e.g., risk ratio, difference in means). | 9-11 |
| Synthesis of results | 14 | Describe the methods of handling data and combining results of studies, if done, including measures of consistency (e.g., I^2^) for each meta-analysis. | 9-11 |

| **Section/topic** | **#** | **Checklist item** | **Reported on page/Table/Figure** |
| --- | --- | --- | --- |
| Risk of bias across studies | 15 | Specify any assessment of risk of bias that may affect the cumulative evidence (e.g., publication bias, selective reporting within studies). | 9, 11, Supp Table 3 |
| Additional analyses | 16 | Describe methods of additional analyses (e.g., sensitivity or subgroup analyses, meta-regression), if done, indicating which were pre-specified. | 10, 11 |
| **RESULTS** | | |  |
| Study selection | 17 | Give numbers of studies screened, assessed for eligibility, and included in the review, with reasons for exclusions at each stage, ideally with a flow diagram. | 12, Figure 1 |
| Study characteristics | 18 | For each study, present characteristics for which data were extracted (e.g., study size, PICOS, follow-up period) and provide the citations. | Table 1, Supp Table 2 |
| Risk of bias within studies | 19 | Present data on risk of bias of each study and, if available, any outcome level assessment (see item 12). | 25, Supp Table 3 |
| Results of individual studies | 20 | For all outcomes considered (benefits or harms), present, for each study: (a) simple summary data for each intervention group (b) effect estimates and confidence intervals, ideally with a forest plot. | Supp Table 2, Figures 2-4/Supp Figs |
| Synthesis of results | 21 | Present the main results of the review. If meta-analyses are done, include for each, confidence intervals and measures of consistency. | 15-25, Supp Table 2, Figures 2-4/Supp Figs |
| Risk of bias across studies | 22 | Present results of any assessment of risk of bias across studies (see Item 15). | Supp Table 3, Supp Figs |
| Additional analysis | 23 | Give results of additional analyses, if done (e.g., sensitivity or subgroup analyses, meta-regression [see Item 16]). | Supp Figs |
| **DISCUSSION** | | |  |
| Summary of evidence | 24 | Summarize the main findings including the strength of evidence for each main outcome; consider their relevance to key groups (e.g., healthcare providers, users, and policy makers). | 25-27 |
| Limitations | 25 | Discuss limitations at study and outcome level (e.g., risk of bias), and at review-level (e.g., incomplete retrieval of identified research, reporting bias). | 27, 28 |
| Conclusions | 26 | Provide a general interpretation of the results in the context of other evidence, and implications for future research. | 28-31 |
| **FUNDING** | | |  |
| Funding | 27 | Describe sources of funding for the systematic review and other support (e.g., supply of data); role of funders for the systematic review. | 31 |

***Supplementary Table 2****. GMT ratios and vaccine immunogenicity data*

|  | **Study ^ref^ (year)** | **Acute immune response^†^** | | | **Antibody persistence^‡^** | | | **Transplacental antibodies**^§^ | | | **Seroprotection & seroconversion rates**^¶^ |
| --- | --- | --- | --- | --- | --- | --- | --- | --- | --- | --- | --- |
|  |  | 2^nd^ : 1^st^ | 3^rd^ : 2^nd^ | 3^rd^ : 1^st^ | 2^nd^ : 1^st^ | 3^rd^ : 2^nd^ | 3^rd^ : 1^st^ | 2^nd^ : 1^st^ | 3^rd^ : 2^nd^ | 3^rd^ : 1^st^ |  |
| **Seasonal epidemics** | Kostinov  et al.[^47^](#_ENREF_47) (2015) | - | H1N1: 1.08 [0.49, 2.39]  H3N2: 2.63 [1.31, 5.29]  B: 1.47 [0.49, 4.36] | - | - | - | - | - | H1N1: 1.57 [0.77, 3.20]  H3N2: 1.99 [1.11, 3.57]  B: 1.20 [0.61, 2.36] | - | sc 1 month post-vaccination:  2^nd^ TRI: H1N1: 65, H3N2: 30, B: 52;  3^rd^ TRI: H1N1: 73, H3N2: 73*, B: 82*  sp 2-3 days post-delivery (in mother):  2^nd^ TRI: H1N1: 79, H3N2: 63, B: 84;  3^rd^ TRI: H1N1: 86*, H3N2: 76*, B: 95  (no difference at 3 or 6 months)  sp 2-3 days post-delivery (infant):  2^nd^ TRI: H1N1: 39, H3N2: 37, B: 63;  3^rd^ TRI: H1N1: 68*, H3N2: 76*, B: 74  (decreased at 3 months post-delivery in both, but 3^rd^ TRI* still higher)  sp 6 months post-delivery (in mother):  2^nd^ TRI: H1N1: 55^, H3N2: 30^, B: 70^;  3^rd^ TRI: H1N1: 67^, H3N2: 52^, B: 67^ |
|  | Madhi et al.[^44^](#_ENREF_44) (2014) | - | H1N1: 1.90 [1.12, 3.23]  H3N2: 1.15 [0.66, 2.00]  B: 1.17 [0.76, 1.80] | - | - | H1N1: 0.80 [0.46, 1.38]  H3N2: 1.56 [0.78, 3.10]  B: 1.07 [0.64, 1.80] | - | - | H1N1: 0.97 [0.55, 1.71]  H3N2: 0.99 [0.54, 1.81]  B: 1.02 [0.65, 1.61] | - | sc^ 1 month post-vaccination:  H1N1: 73 [64, 80], H3N2: 65 [56, 73], B: 92 [87, 96];  sp^ 1 month post-vaccination:  H1N1: 94 [88, 97], H3N2: 79 [71, 85], B: 97 [93, 99]  sp^ >60 against all strains for newborns in 1^st^ week of life |
|  | Blanchard-Rohner  et al.[^48^](#_ENREF_48) (2013) | - | - | - | - | - | - | H1N1: 1.09 [0.34, 3.46]  H3N2: 1.49 [0.50, 4.41]  B: 2.89  [1.38, 6.06] | H1N1: 0.66 [0.35, 1.24]  H3N2: 0.87 [0.46, 1.64]  B: 0.72 [0.42, 1.25] | H1N1: 0.71 [0.22, 2.26]  H3N2: 1.30 [0.45, 3.73]  B: 2.09 [1.00, 4.39] | sp^ in cord-blood:  H1N1: 86, H3N2: 84, B: 85  sp in cord-blood (H1N1, H3N2, B, respectively):  <15 days pre-delivery: 67, 33, 50 (OR* p>0.05);  15-30 days pre-delivery: 79, 86, 93;  31-90 days pre-delivery: 91, 94, 88;  91-120 days pre-delivery: 91, 91, 91;  >121 days pre-delivery: 87, 88, 86 |
|  | Christian  et al.[^49^](#_ENREF_49) (2013) | H1N1: 2.14 [0.07,69.24]  H3N2: 1.48 [0.39, 5.59]  B: 4.07 [1.30,12.73] | H1N1: 3.39 [0.62,18.62]  H3N2: 1.66 [0.43, 6.41]  B: 2.95 [1.02, 8.52] | H1N1: 7.24 [0.18,291.58]  H3N2: 2.46 [0.52,11.62]  B: 12.02 [3.45,41.88] | - | - | - | - | - | - | sc^ 1 month post-vaccination:  H1N1: 70, H3N2: 63, B: 63  sp^ 1 month post-vaccination:  H1N1: 89, H3N2: 81, B: 83 |
|  |  |  |  |  |  |  |  |  |  |  |  |
|  | Garcia-Putnam  et al.[^46^](#_ENREF_46) (2013) | - | H1N1: 2.91 [0.50,16.76]  H3N2: 6.18 [1.40,27.27]  B: 2.60 [0.60,11.18] | - | - | H1N1: 0.90 [0.14, 5.82]  H3N2: 1.06 [0.20, 5.70]  B: 1.46 [0.36, 5.86] | - | - | H1N1: 2.12 [0.42,10.68]  H3N2: 3.00 [0.87,10.36]  B: 1.77 [0.67, 4.69] | - | sp 4 weeks post-vaccination:  2^nd^ TRI: H1N1: 75; 3^rd^ TRI: H1N1: 100  sp at delivery (in mother):  2^nd^ TRI: H1N1: 92  GMT 6 weeks postpartum:  3^rd^ TRI: H3N2 comparatively higher (p<0.02) |
|  | Lin et al.[^36^](#_ENREF_36) (2013) | - | H1N1: 0.56 [0.24, 1.30]  H3N2: 1.55 [0.56, 4.29]  B: 1.28 [0.51, 3.19] | - | - | H1N1: 1.97 [0.78, 4.96]  H3N2: 1.59 [0.47, 5.39]  B: 0.96 [0.35, 2.66] | - | - | H1N1: 2.17 [0.94, 5.00]  H3N2: 3.06 [1.28, 7.34]  B: 1.67 [0.70, 3.98] | - | sc^ 28 days post-vaccination:  H1N1: 67, H3N2: 63, B: 44  sp^ 28 days post-vaccination:  H1N1: 91, H3N2: 85, B: 57  sc^ at delivery (in mother):  H1N1: 64, H3N2: 50, B: 41  sp^ at delivery (in mother):  H1N1: 86, H3N2: 68, B: 48  sp^ in cord-blood:  H1N1: 88, H3N2: 71, B: 48 |
|  | Schlaudecker  et al.[^50^](#_ENREF_50) (2012) | H1N1: 1.49 [0.37, 6.05]  H3N2: 1.40 [0.59, 3.31]  B: 1.23 [0.20, 7.49] | H1N1: 1.06 [0.24, 4.77]  H3N2: 0.95 [0.30, 2.99]  B: 0.96 [0.24, 3.93] | H1N1: 1.59 [0.23, 1.17]  H3N2: 1.33 [0.40, 4.46]  B: 1.19 [0.11, 2.63] | - | - | - | - | - | - | sc^ 28 days post-vaccination:  H1N1: 41 [25, 59], H3N2: 10 [0, 21], B: 21 [6, 35]  sp^ 28 days post-vaccination:  H1N1: 100, H3N2: 100, B: 59 [41, 77] |
|  | Eick et al.[^51^](#_ENREF_51) (2011) | - | - | - | - | - | - | - | H1N1: 1.28 [0.95, 1.72]  H3N2: 1.13 [0.83,1.54]; 1.28  [0.92, 1.77]  B: 1.16 [0.86,1.57]; 1.13  [0.74, 1.71] | - | sp in cord-blood:  2^nd^ TRI: H1N1: 61, H3N2: 80; 65, B: 30.4; 67;  3^rd^ TRI: H1N1: 70, H3N2: 78; 69, B: 43; 71  (p>0.05 for all b/n TRI comparisons) |
|  | Yamaguchi  et al.[^38^](#_ENREF_38) (2009) | - | H1N1: 1.42 [0.77, 2.63]  H3N2: 1.03 [0.54, 1.96]  B: 1.15 [0.55, 2.40] | - | - | H1N1: 0.78 [0.43, 1.41]  H3N2: 0.88 [0.48, 1.60]  B: 0.65 [0.36, 1.17] | - | - | H1N1: 1.54 [0.90, 2.64]  H3N2: 1.05 [0.65, 1.69]  B: 1.38 [0.93, 2.04] | - | sc^ 1 month post-vaccination:  H1N1: 51, H3N2: 28, B: 40  Maintenance of elevated HI titre: 2^nd^ TRI: 66 ± 27;  3^rd^ TRI: 94 ± 31 (p<0.001)  Transplacental transfer rate: 2^nd^ TRI: 161 ± 71;  3^rd^ TRI: 127 ± 85 (p=0.02) |
|  |  |  |  |  |  |  |  |  |  |  |  |
| **2009 pandemic** | Bischoff  et al.[^52^](#_ENREF_52) (2013) | - | 15µg U/A: 2.34  [0.05,118.75]  7.5µg F/A: 0.65  [0.01,60.21]  3.75µg H/A: 4.10 [0.02,887.73] | - | - | - | - | - | - | - | sc^ 3 weeks post-vaccination:  15µg U/A: 83 [67, 93], 7.5µg F/A: 95 [82, 99], 3.75µg H/A: 86 [57, 98]  sc^ 3 months post-vaccination:  15µg U/A: 74 [58, 87], 7.5µg F/A: 70 [51, 85], 3.75µg H/A: 64 [37, 87]  sc^ 10 months post-vaccination:  15µg U/A: 70 [51, 84], 7.5µg F/A: 59 [39, 76]*, 3.75µg H/A: 55 [23, 83]  sp^ 3 weeks post-vaccination:  15µg U/A: 88 [77, 95], 7.5µg F/A: 96 [88, 100], 3.75µg H/A: 89 [72, 98]  sp^ 3 months post-vaccination:  15µg U/A: 76 [63, 86], 7.5µg F/A: 82 [68, 91], 3.75µg H/A: 74 [54, 89]  sp^ 10 months post-vaccination:  15µg U/A: 74 [60, 85], 7.5µg F/A: 70 [55, 83]*, 3.75µg H/A: 67 [45, 84] |
|  | Chao et al.[^43^](#_ENREF_43) (2013) | - | - | - | - | - | - | U/A: 1.62  [0.89, 2.94]  Adj: 1.22  [0.62, 2.39] | U/A: 0.57 [0.30, 1.08]  ~ | U/A: 0.92 [0.40, 2.09]  ~ | sp^ at delivery (in mother): 78  sp^ in cord-blood: 76 |
|  | Fisher et al.[^42^](#_ENREF_42) (2012) | - | - | - | - | - | - | 2.88  [0.36, 22.88] | 3.00  [0.09,95.79] | 8.62  [0.38,194.16] | Mean HI titre at delivery (in mother) >1:40  Significant linear decline in HI titres b/n vaccination and delivery (p=0.009) |
|  | Horiya et al.[^35^](#_ENREF_35) (2011) | 1^st^ D: 0.87 [0.33, 2.26]  2^nd^ D: 1.16 [0.44, 3.08] | 1^st^ D: 0.90 [0.40, 2.02]  2^nd^ D: 0.98 [0.44, 2.17] | 1^st^ D: 0.78 [0.25, 2.40]  2^nd^ D: 1.13 [0.37, 3.46] | 1^st^ D: 1.59 [0.56, 4.54]  2^nd^ D: 1.19 [0.41, 3.49] | 1^st^ D: 1.16 [0.53, 2.54]  2^nd^ D: 1.07 [0.50, 2.28] | 1^st^ D: 1.86 [0.60, 5.76]  2^nd^ D: 1.28 [0.40, 4.05] | ~  2 D: 1.59  [0.75, 3.38] | 1 D: 0.98 [0.52, 1.88]  2 D: 1.26 [0.69, 2.32] | ~  2 D: 2.01 [0.84, 4.82] | sc 3 weeks post-vaccination:  1^st^ dose: 1^st^ TRI: 87, 2^nd^ TRI: 86, 3^rd^ TRI: 96;  2^nd^ dose: 1^st^ TRI: 87, 2^nd^ TRI: 87, 3^rd^ TRI: 96  HI titres at delivery (in mother) and in cord-blood: non-significantly higher for women vaccinated in later stages of pregnancy and for women who received 2 doses |
|  | Jackson  et al.[^41^](#_ENREF_41) (2011) | - | 25µg: 2.22 [0.93, 5.31]; 1.79  [0.67, 4.79]  49µg: 1.28 [0.52, 3.14]; 0.94  [0.31, 2.90] | - | - | 25µg: 0.96 [0.30,3.08]; 1.17  [0.33, 4.17]    49µg: 2.85 [0.60,13.52]; 3.69  [0.68, 0.15] | - | - | 25µg: 1.67 [0.67, 4.16]  49µg: 2.91 [1.17, 7.23] | - | sc^ 3 weeks post-vaccination:  25µg: 89 [78, 96], 95 [82, 99];  49µg: 97 [88, 100], 92 [81, 98]  sp^ 3 weeks post-vaccination:  25µg: 93 [82, 98], 95 [82, 99];  49µg: 97 [88, 100], 92 [81, 98]  sp^ at delivery (in mother):  25µg: 85 [71, 94];  49µg: 62 [46, 75]  sp^ in cord-blood:  25µg: 87 [73, 96];  49µg: 89 [76, 96]  Longer vac to delivery interval: lower HI titres at delivery (p<0.05) |
|  | Ohfuji et al.[^37^](#_ENREF_37) (2011) | 1^st^ D: 1.29 [0.61, 2.70]  2^nd^ D: 1.16 [0.59, 2.29] | 1^st^ D: 1.13 [0.66, 1.93]  2^nd^ D: 1.50 [0.92, 2.43] | 1^st^ D: 1.45 [0.73, 2.90]  2^nd^ D: 1.74 [0.91, 3.35] |  | - | - | - | - | - | sc 3 weeks post 1^st^ dose:  1^st^ TRI: 92, 2^nd^ TRI: 82, 3^rd^ TRI: 92  sc 4 weeks post 2^nd^ dose:  1^st^ TRI: 76, 2^nd^ TRI: 91, 3^rd^ TRI: 94 (p=0.02)  sp 3 weeks post 1^st^ dose:  1^st^ TRI: 85, 2^nd^ TRI: 87, 3^rd^ TRI: 91  sp 4 weeks post 2^nd^ dose:  1^st^ TRI: 92, 2^nd^ TRI: 84, 3^rd^ TRI: 94 |
|  | Tsatsaris  et al.[^53^](#_ENREF_53) (2011) | - | 0.87  [0.43, 1.80] | - | - | 1.23  [0.57, 2.66] | - | - | 1.15  [0.59, 2.23] | - | sc 21 days post-vaccination:  2^nd^ TRI: 95 [85, 99], 3^rd^ TRI: 91 [79, 98]  sp 21 days post-vaccination:  2^nd^ TRI: 98 [90, 100], 3^rd^ TRI: 98 [88, 100]  sp at delivery (in mother):  2^nd^ TRI: 92 [81, 98], 3^rd^ TRI: 91 [80, 98]  sp 3 months post-vaccination:  2^nd^ TRI: 94 [82, 99], 3^rd^ TRI: 86 [71, 95]  sp in cord-blood:  2^nd^ TRI: 96 [85, 99], 3^rd^ TRI: 95 [83, 99]  (p>0.05 for all b/n TRI comparisons) |

***^†^*** *Bischoff et al. U/A: unadjuvanted, F/A: full adjuvanted, H/A: half adjuvanted; Horiya et al. & Ohfuji et al. ratios presented for post 1^st^ dose & post 2^nd^ dose; Jackson et al. ratios presented for post 1^st^ dose; post 2^nd^ dose.*

***^‡^*** *Horiya et al. & Ohfuji et al. ratios presented for post 1^st^ dose; post 2^nd^ dose; Jackson et al. ratios presented for post 1^st^ dose; post 2^nd^ dose.*

***^§^*** *Eick et al. ratios presented for two unique H3N2 and B strains, respectively; Chao et al. U/A: unadjuvanted, Adj: adjuvanted; Chao et al. & Horiya et al. ~: no ratios as n=1; Horiya et al. 1 D: one dose group, 2 D: two dose group.*

***^¶^*** *sp (seroprotection) rate: the percentage (to nearest whole number) of subjects with a post-vaccination hemagglutination inhibition (HI) titre ≥1:40; sc (seroconversion) rate, the percentage (to nearest whole number) of subjects with a post-vaccination HI titre ≥1:40 who had a pre-vaccination HI titre of <1:40; TRI, trimester; Kostinov et al. *: p<0.01 b/n TRIs, ^: p<0.01 compared with 1 month post-vaccination; sc^ & sp^: proportions not stratified by TRI; Blanchard-Rohner et al.: stratified by vaccination-delivery interval, *: odds ratio for seroprotection compared with non-vaccinated women; Eick et al. two unique H3N2 and B strains; Yamaguchi et al. maintenance of elevated HI titre: HI titre in maternal serum at delivery relative to 1 month post-vaccination (%), transplacental transfer rate: HI titre in cord-blood relative to maternal serum at delivery (%); Bischoff et al. *: significant decrease over time; Fisher et al. linear regression of maternal HI titre and days from vaccination to blood-draw; Jackson et al. proportions presented for post 1^st^ dose; post 2^nd^ dose; other notes: where possible, results are presented with 95% confidence intervals in square brackets; Garcia-Putnam et al. was only identified as a published abstract, limiting data availability*

***Supplementary Table 3.*** *Summary of risk of bias assessment for included studies.*

|  |  | Risk of bias judgements | |  |
| --- | --- | --- | --- | --- |
|  | Study^ref^ | Domains | Overall | Notes |
| Randomised  (RoB 2.0) | Bischoff et al.^52^ | 1. Low  2. Low  3. Low  4. Low  5. Low | Low | - |
|  | Jackson et al.^41^ | 1. Low  2. Low  3. Some concerns  4. Low  5. Low | Some concerns | Concerns due to missing data (loss to follow-up) for the mother at delivery and cord-blood outcomes in the 49mg dose group (all other outcomes have low bias). |
|  | Madhi et al.^44^ | 1. Low  2. Low  3. Some concerns  4. Low  5. Low | Some concerns | Some concerns due to missing data (loss to follow-up) for the mother at delivery (all other outcomes have low bias). |
| Non-randomised  (ROBINS-I) | Blanchard-Rohner et al.^48^ | 1. Low  2. Low  3. Low  4. Low  5. Low  6. Low  7. Low | Low | - |
|  | Chao et al.^43^ | 1. Low  2. Low  3. Low  4. Low  5. Low  6. Low  7. Low | Low | Very low numbers in adjuvanted vaccine group in 2^nd^ (n=2) and 3^rd^ (n=1, not included in analysis) trimesters. |
|  | Christian et al.^49^ | 1. Low  2. Low  3. Low  4. Low  5. Low  6. Low  7. Low | Low | - |
|  | Eick et al.^51^ | 1. Moderate  2. Low  3. Low  4. Low  5. Low  6. Low  7. Low | Low | Although confounding may be an issue, any effect should be equal in both women vaccinated in the 2^nd^ and 3^rd^ trimesters – furthermore, the outcome measure is a ratio of GMTs. |
|  | Fisher et al.^42^ | 1. Low  2. Low  3. Low  4. Low  5. Low  6. Low  7. Low | Low | Some concern over extremely low sample size (1^st^ TRI: 7, 2^nd^ TRI: 3, 3^rd^ TRI: 4) |
|  | Garcia-Putnam et al.^46^ | 1. Low  2. Low  3. Low  4. Low  5. Low  6. Low  7. Low | Low | Data taken from conference abstract |
|  | Horiya et al.^35^ | 1. Low  2. Low  3. N/A  4. Low  5. Low  6. N/A  7. Low | Low | Single-arm study |
|  | Kostinov et al.^47^ | 1. Low  2. Low  3. Low  4. Low  5. Low  6. Low  7. Low | Low | - |
|  | Lin et al.^36^ | 1. Low  2. Low  3. N/A  4. Low  5. Low  6. N/A  7. Low | Low | Single-arm study |
|  | Ohfuji et al.^37^ | 1. Low  2. Low  3. N/A  4. Low  5. Low  6. N/A  7. Low | Low | Single-arm study |
|  | Schlaudecker et al.^50^ | 1. Low  2. Low  3. Low  4. Low  5. Low  6. Low  7. Low | Low | - |
|  | Tsatsaris et al.^53^ | 1. Low  2. Low  3. Low  4. Low  5. Low  6. Low  7. Low | Low | - |
|  | Yamaguchi et al.^38^ | 1. Low  2. Low  3. N/A  4. Low  5. Low  6. N/A  7. Low | Low | Single-arm study  Some concerns for maternal fold-change in GMT comparison between trimesters |

Key for Domains:

Randomised (RoB 2.0)

1. Bias arising from the randomization process
2. Bias due to deviations from intended interventions
3. Bias due to missing outcome data
4. Bias in measurement of the outcome
5. Bias in selection of the reported result

Non-randomised (ROBINS-I)

1. Bias due to confounding
2. Bias in selection of participants into the study
3. Bias in classification of interventions
4. Bias due to deviations from intended interventions
5. Bias due to missing data
6. Bias in measurement of outcomes
7. Bias in selection of the reported result


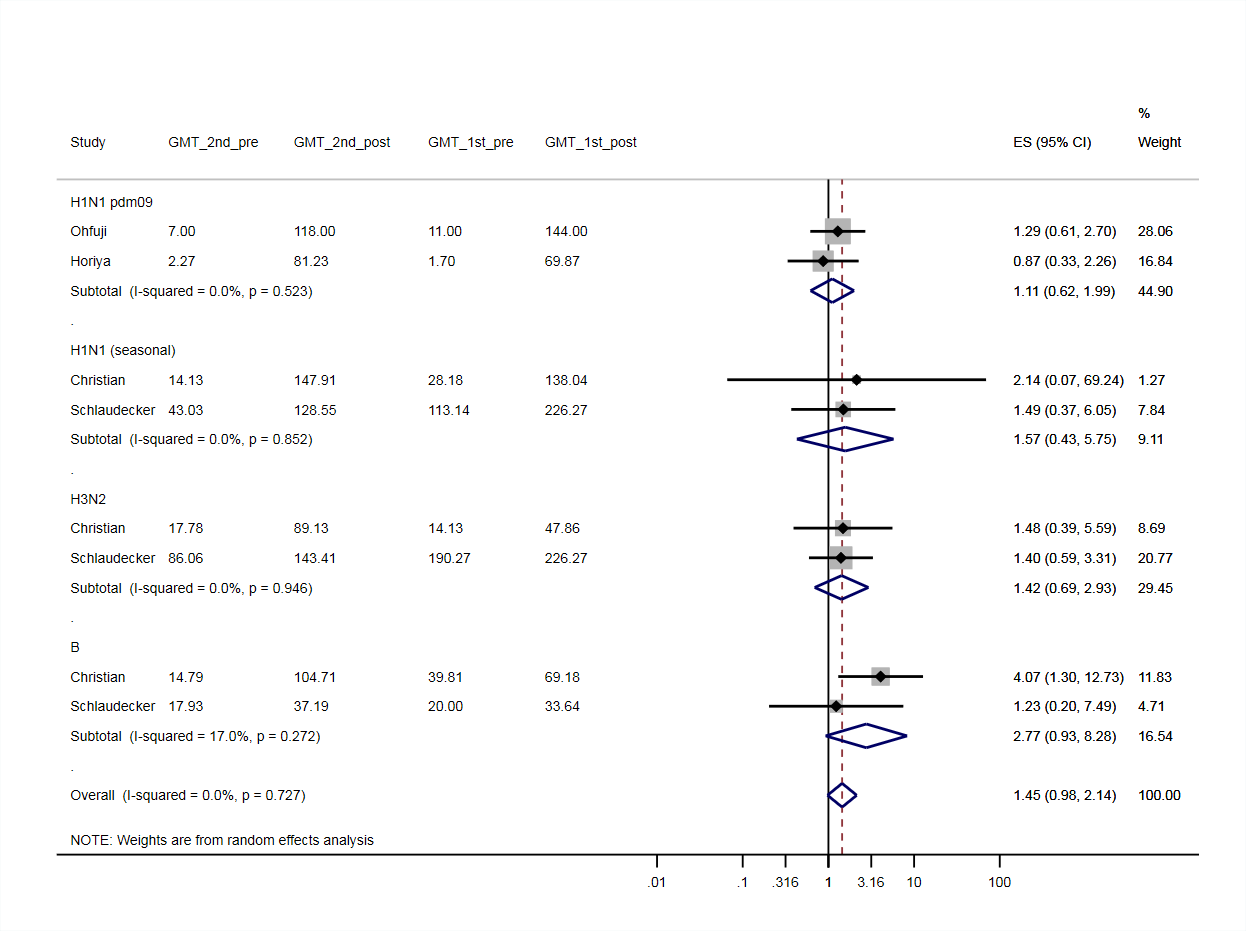


Supplementary Figure 1. A forest plot of the geometric mean fold increase (GMFI) ratio, pre-vaccination to post-vaccination, comparing women vaccinated in the 2^nd^ trimester with women vaccinated in the 1^st^ trimester.

GMT_2^nd^_pre: geometric mean titre (GMT) pre-vaccination, 2^nd^ trimester vaccination

GMT_2^nd^_post: GMT post-vaccination, 2^nd^ trimester vaccination

GMT_1^st^_pre: GMT pre-vaccination, 1^st^ trimester vaccination

GMT_1^st^_post: GMT post-vaccination, 1^st^ trimester vaccination

ES (95% CI): Effect size (GMFI) (95% confidence interval)


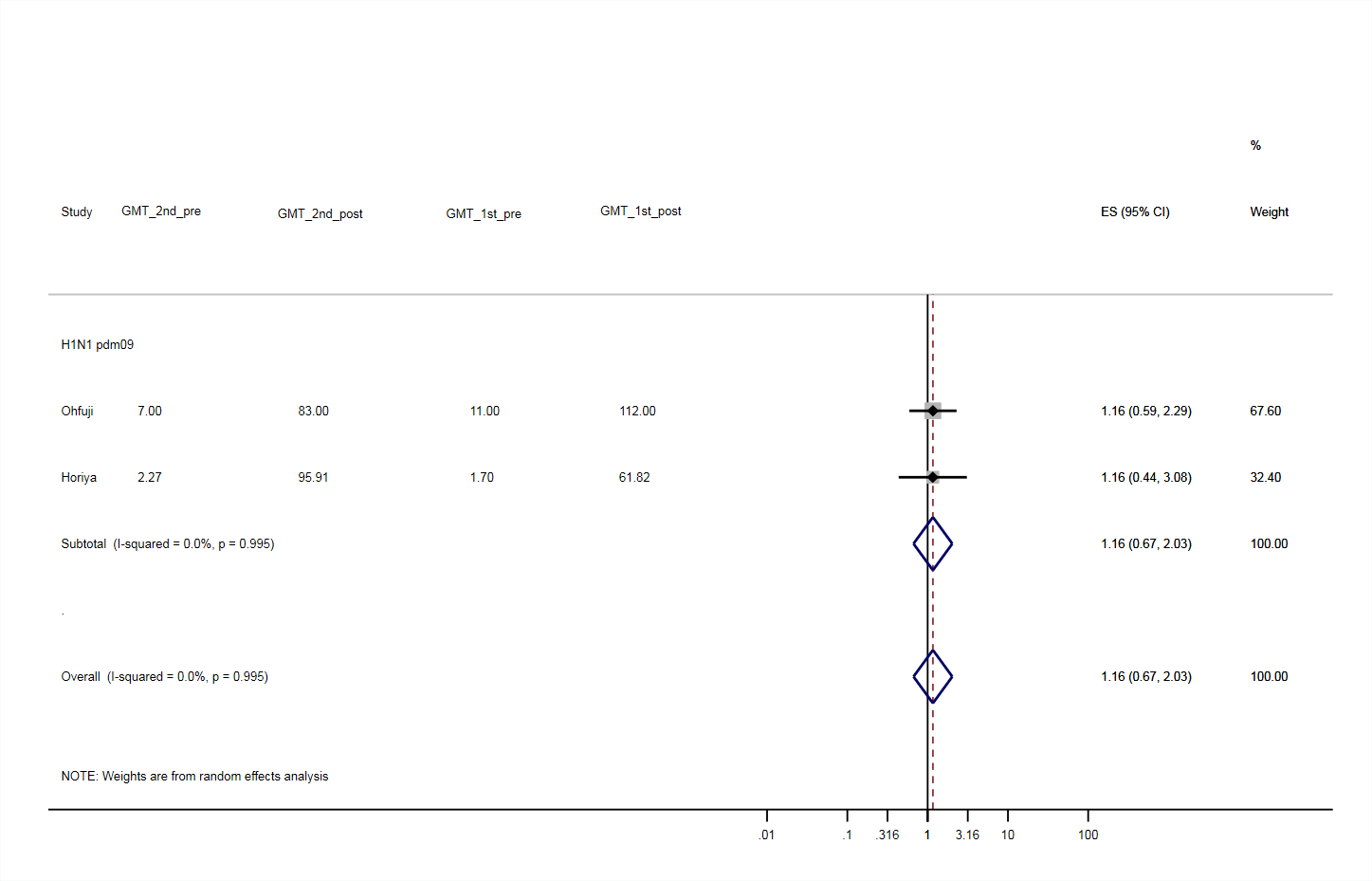


Supplementary Figure 2. A forest plot of the geometric mean fold increase (GMFI) ratio, pre-vaccination to post-2^nd^ vaccination, comparing women vaccinated in the 2^nd^ trimester with women vaccinated in the 1^st^ trimester.

GMT_2nd_pre: geometric mean titre (GMT) pre-vaccination, 2nd trimester vaccination

GMT_2nd_post: GMT post-2^nd^ vaccination, 2nd trimester vaccination

GMT_1st_pre: GMT pre-vaccination, 1st trimester vaccination

GMT_1st_post: GMT post-2^nd^ vaccination, 1st trimester vaccination

ES (95% CI): Effect size (GMFI) (95% confidence interval)


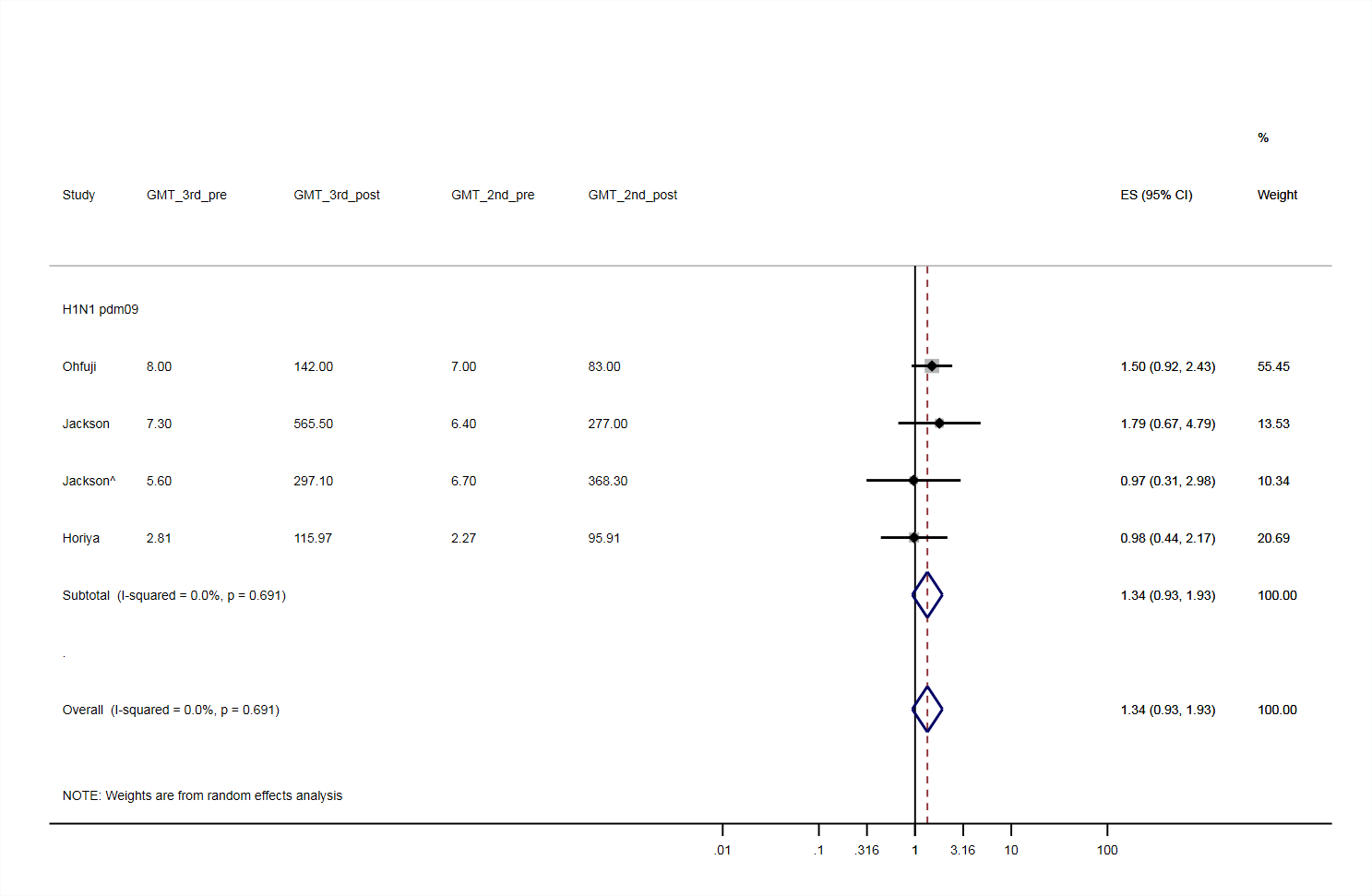


Supplementary Figure 3. A forest plot of the geometric mean fold increase (GMFI) ratio, pre-vaccination to post-2^nd^ vaccination, comparing women vaccinated in the 3^rd^ trimester with women vaccinated in the 2^nd^ trimester.

^ Jackson et al. 49µg group (as opposed to the 25µg group)

GMT_3^rd^_pre: geometric mean titre (GMT) pre-vaccination, 3^rd^ trimester vaccination

GMT_3^rd^_post: GMT post-2^nd^ vaccination, 3^rd^ trimester vaccination

GMT_2^nd^_pre: GMT pre-vaccination, 2^nd^ trimester vaccination

GMT_2^nd^_post: GMT post-2^nd^ vaccination, 2^nd^ trimester vaccination

ES (95% CI): Effect size (GMFI) (95% confidence interval)


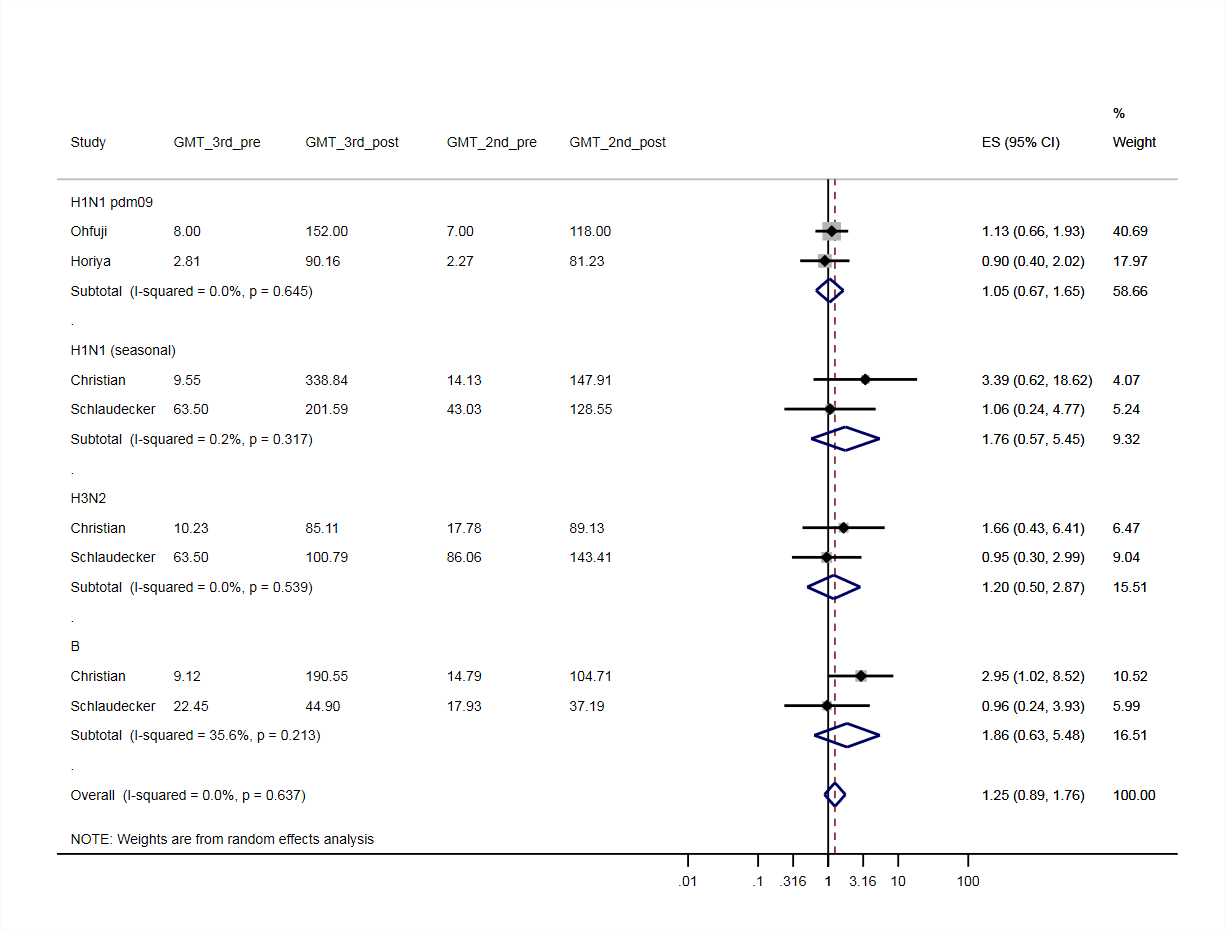


Supplementary Figure 4. A sensitivity analysis including only studies with women vaccinated in the 1^st^ trimester as well as the 3^rd^ and 2^nd^ trimesters; this is a forest plot of the geometric mean fold increase (GMFI) ratio, pre-vaccination to post-vaccination, comparing women vaccinated in the 3^rd^ trimester with women vaccinated in the 2^nd^ trimester.

GMT_3rd_pre: geometric mean titre (GMT) pre-vaccination, 3rd trimester vaccination

GMT_3rd_post: GMT post-vaccination, 3rd trimester vaccination

GMT_2nd_pre: GMT pre-vaccination, 2nd trimester vaccination

GMT_2nd_post: GMT post-vaccination, 2nd trimester vaccination

ES (95% CI): Effect size (GMFI) (95% confidence interval)


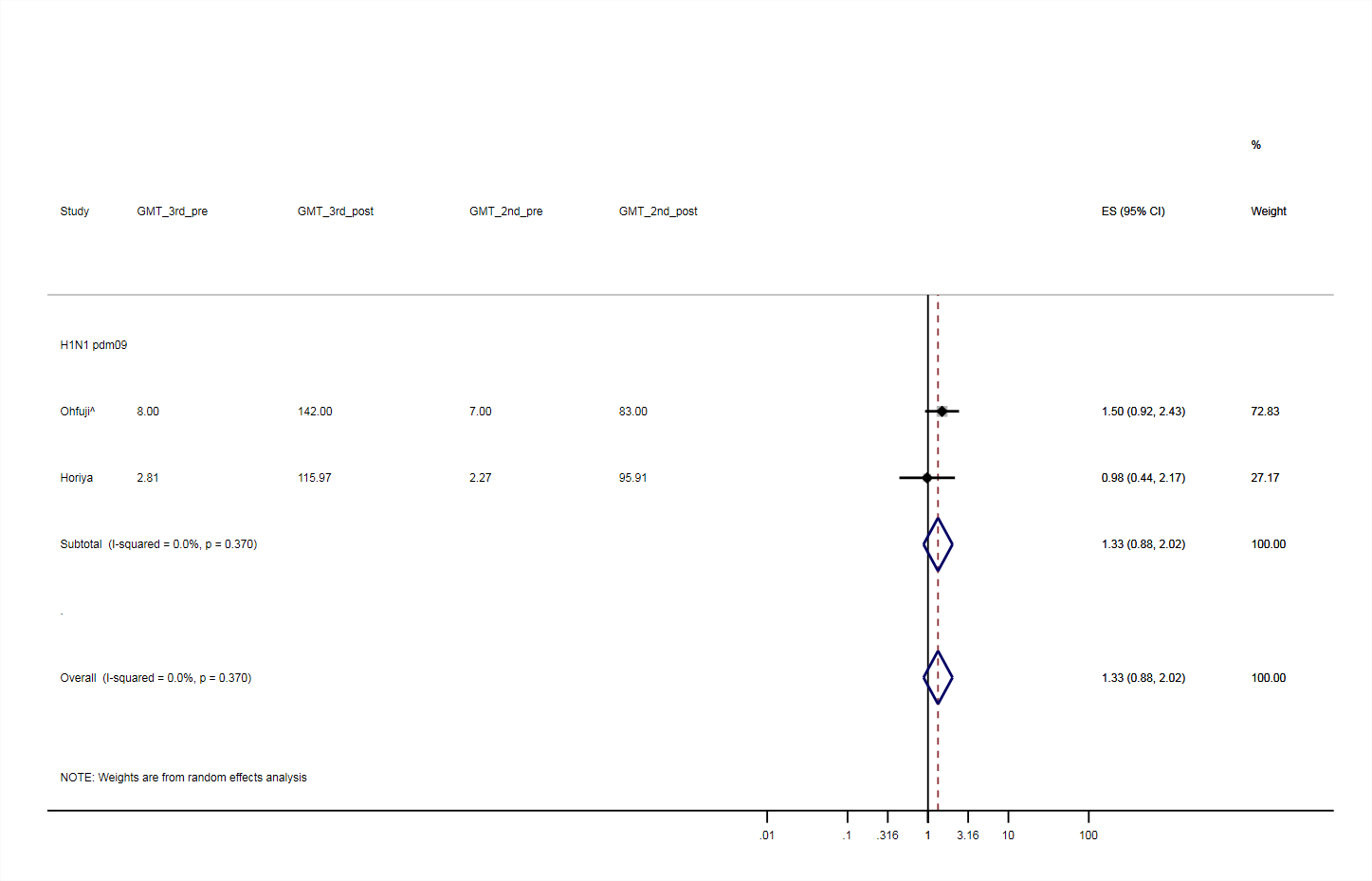


Supplementary Figure 5. A sensitivity analysis including only studies with women vaccinated in the 1^st^ trimester as well as the 3^rd^ and 2^nd^ trimesters; this is a forest plot of the geometric mean fold increase (GMFI) ratio, pre-vaccination to post-2^nd^ vaccination, comparing women vaccinated in the 3^rd^ trimester with women vaccinated in the 2^nd^ trimester

GMT_3rd_pre: geometric mean titre (GMT) pre-vaccination, 3rd trimester vaccination

GMT_3rd_post: GMT post-2^nd^ vaccination, 3rd trimester vaccination

GMT_2nd_pre: GMT pre-vaccination, 2nd trimester vaccination

GMT_2nd_post: GMT post-2^nd^ vaccination, 2nd trimester vaccination

ES (95% CI): Effect size (GMFI) (95% confidence interval)


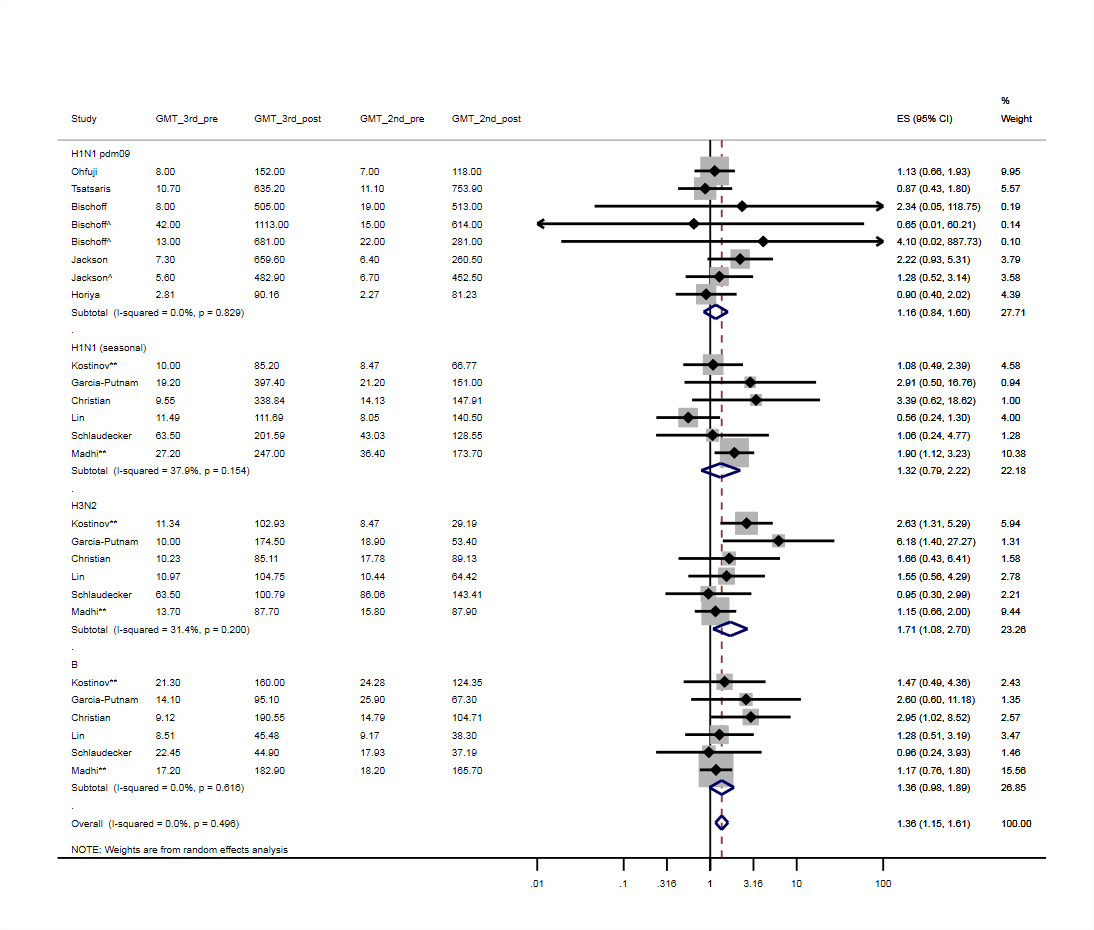


Supplementary Figure 6. A sensitivity analysis that excludes Yamaguchi et al. due to risk of internal bias; this is a forest plot of the geometric mean fold increase (GMFI) ratio, pre-vaccination to post-vaccination, comparing women vaccinated in the 3^rd^ trimester with women vaccinated in the 2^nd^ trimester

^ Bischoff et al. 7.5µg & 3.75µg groups, respectively (as opposed to the 15µg group); Jackson et al. 49µg group (as opposed to the 25µg group)

** Kostinov et al. & Madhi et al. were conducted over two influenza seasons (using same vaccine in both seasons)

GMT_3rd_pre: geometric mean titre (GMT) pre-vaccination, 3rd trimester vaccination

GMT_3rd_post: GMT post-vaccination, 3rd trimester vaccination

GMT_2nd_pre: GMT pre-vaccination, 2nd trimester vaccination

GMT_2nd_post: GMT post-vaccination, 2nd trimester vaccination

ES (95% CI): Effect size (GMFI) (95% confidence interval)


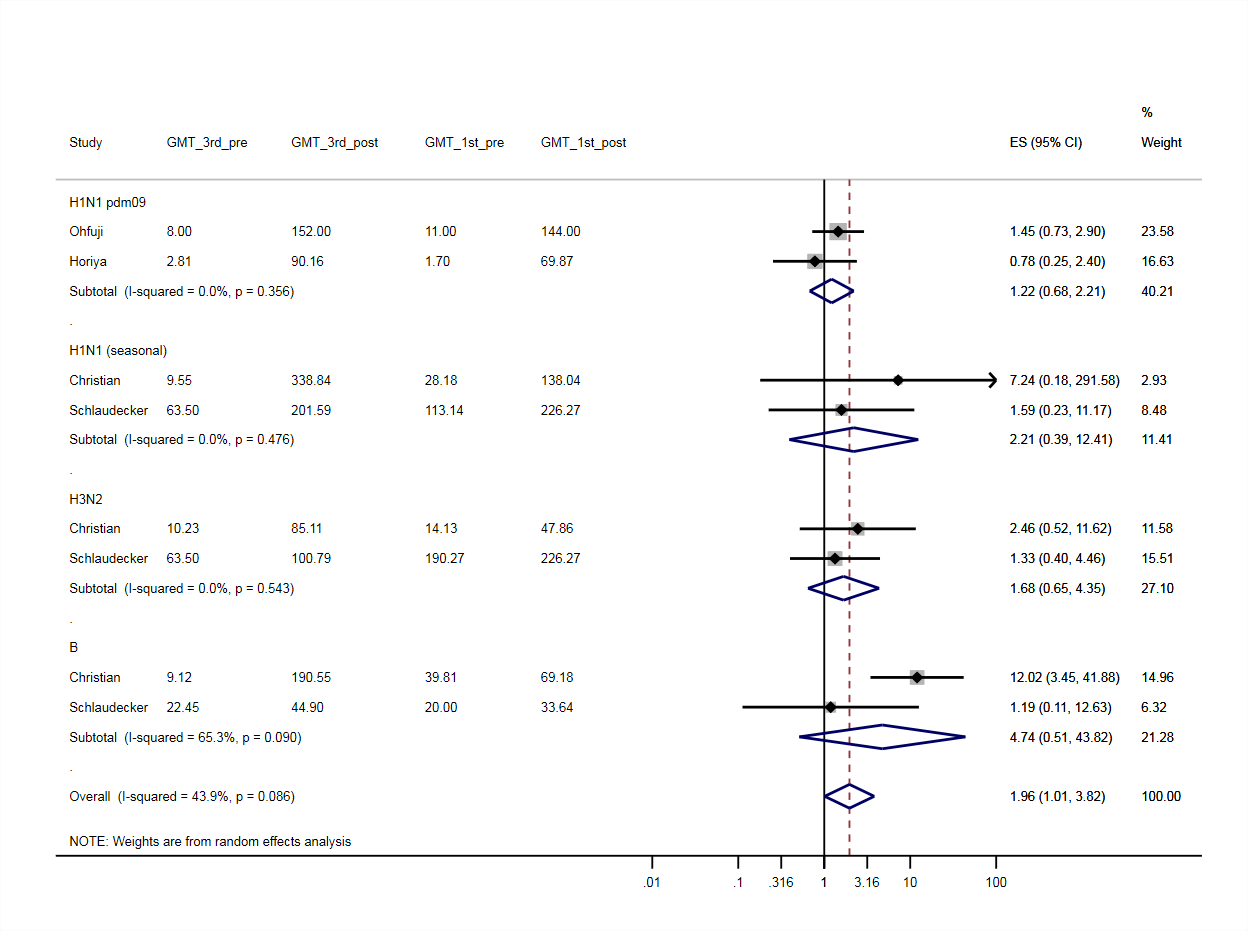


Supplementary Figure 7. A forest plot of the geometric mean fold increase (GMFI) ratio, pre-vaccination to post-vaccination, comparing women vaccinated in the 3^rd^ trimester with women vaccinated in the 1^st^ trimester.

GMT_3^rd^_pre: geometric mean titre (GMT) pre-vaccination, 3^rd^ trimester vaccination

GMT_3^rd^_post: GMT post-vaccination, 3^rd^ trimester vaccination

GMT_1^st^_pre: GMT pre-vaccination, 1^st^ trimester vaccination

GMT_1^st^_post: GMT post-vaccination, 1^st^ trimester vaccination

ES (95% CI): Effect size (GMFI) (95% confidence interval)


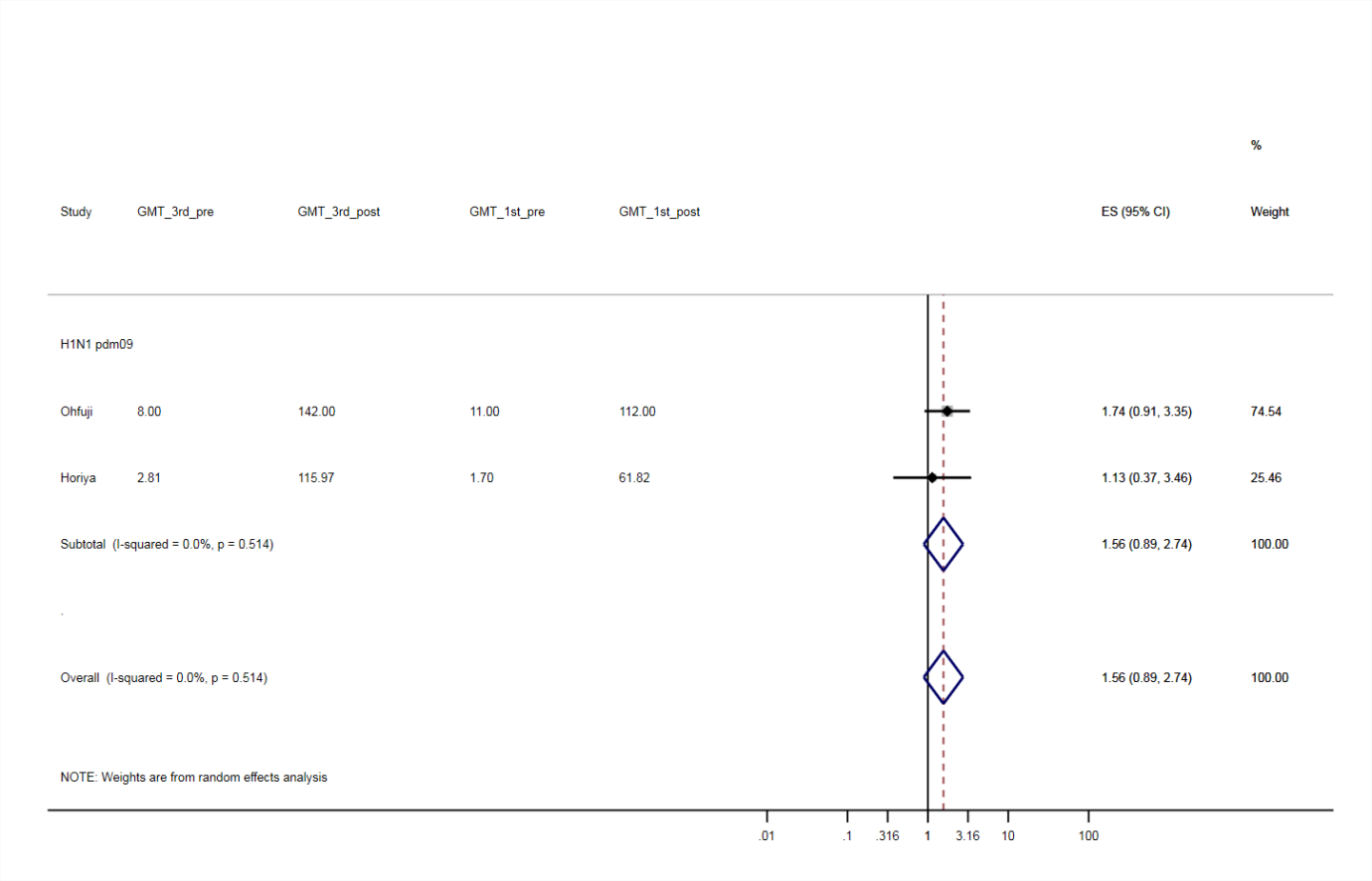


Supplementary Figure 8. A forest plot of the geometric mean fold increase (GMFI) ratio, pre-vaccination to post-2^nd^ vaccination, comparing women vaccinated in the 3^rd^ trimester with women vaccinated in the 1^st^ trimester.

GMT_3rd_pre: geometric mean titre (GMT) pre-vaccination, 3rd trimester vaccination

GMT_3rd_post: GMT post-2^nd^ vaccination, 3rd trimester vaccination

GMT_1st_pre: GMT pre-vaccination, 1st trimester vaccination

GMT_1st_post: GMT post-2^nd^ vaccination, 1st trimester vaccination

ES (95% CI): Effect size (GMFI) (95% confidence interval)


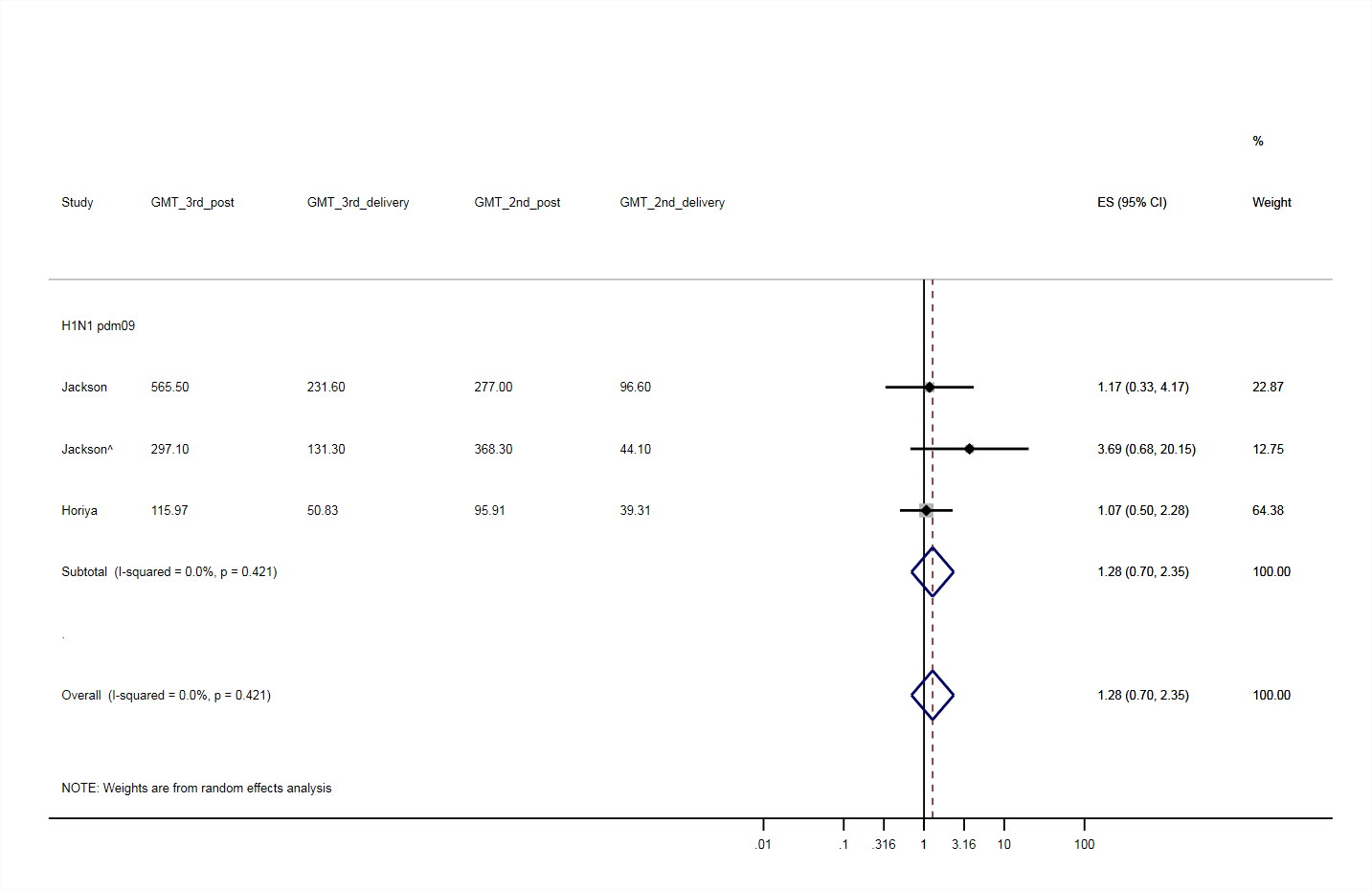


Supplementary Figure 9. A forest plot of the geometric mean fold decrease (GMFD) ratio, post-2^nd^ immunisation to delivery, comparing women vaccinated in the 3rd trimester with women vaccinated in the 2nd trimester.

^ Jackson et al. 49µg group (as opposed to the 25µg group)

GMT_3rd_ post: geometric mean titre (GMT) post-2^nd^ vaccination, 3rd trimester vaccination

GMT_3rd_delivery: GMT in mother at delivery, 3rd trimester vaccination

GMT_2nd_ post: GMT post-2^nd^ vaccination, 2nd trimester vaccination

GMT_2nd_delivery: GMT in mother at delivery, 2nd trimester vaccination

ES (95% CI): Effect size (GMFD) (95% confidence interval)


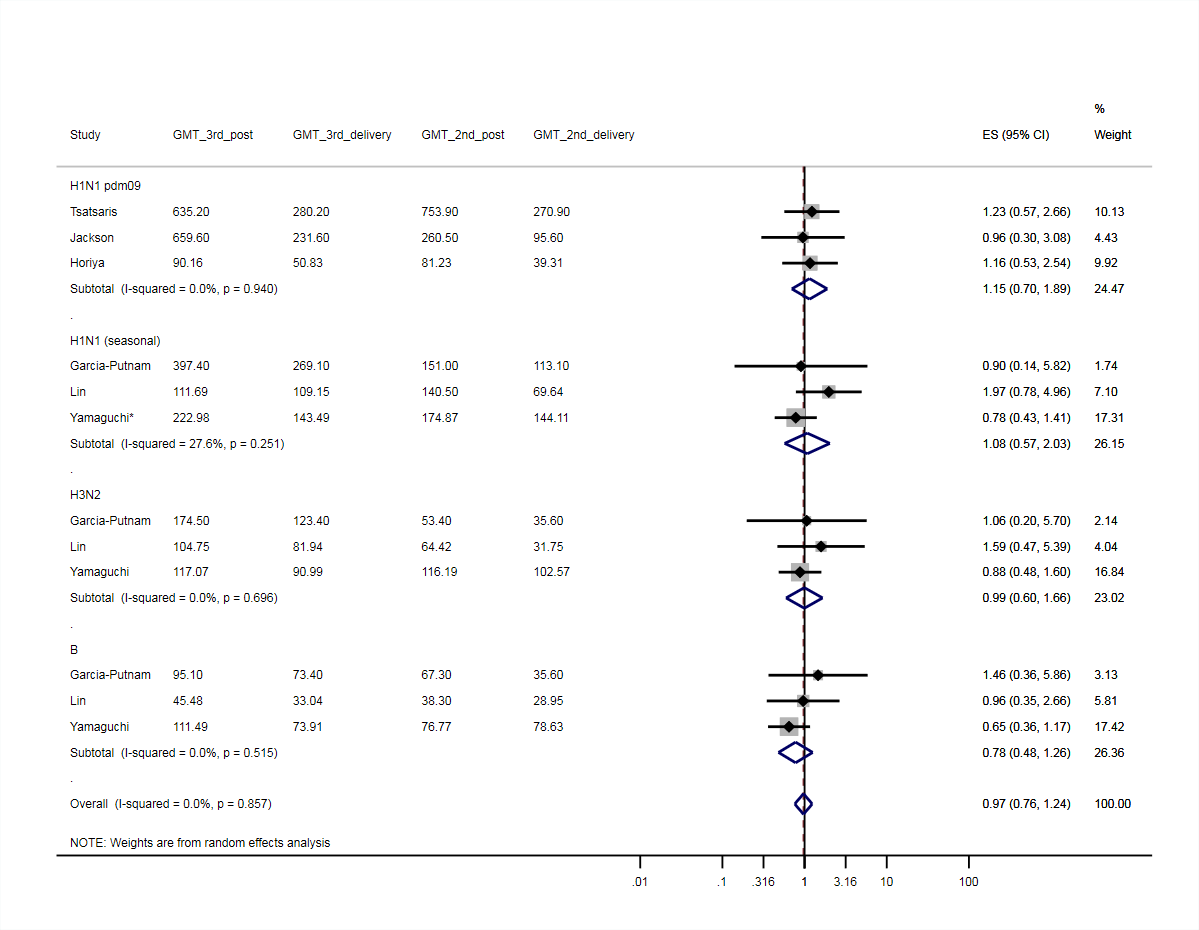


Supplementary Figure 10. A sensitivity analysis that excludes Jackson et al. 49µg group due to risk of internal bias; this is a forest plot of the geometric mean fold decrease (GMFD) ratio, post-immunisation to delivery, comparing women vaccinated in the 3^rd^ trimester with women vaccinated in the 2^nd^ trimester.

* Yamaguchi et al. studied a non-pdm09 H1N1 strain (2007/08 season)

GMT_3rd_ post: geometric mean titre (GMT) post-vaccination, 3rd trimester vaccination

GMT_3rd_delivery: GMT in mother at delivery, 3rd trimester vaccination

GMT_2nd_ post: GMT post-vaccination, 2nd trimester vaccination

GMT_2nd_delivery: GMT in mother at delivery, 2nd trimester vaccination

ES (95% CI): Effect size (GMFD) (95% confidence interval)


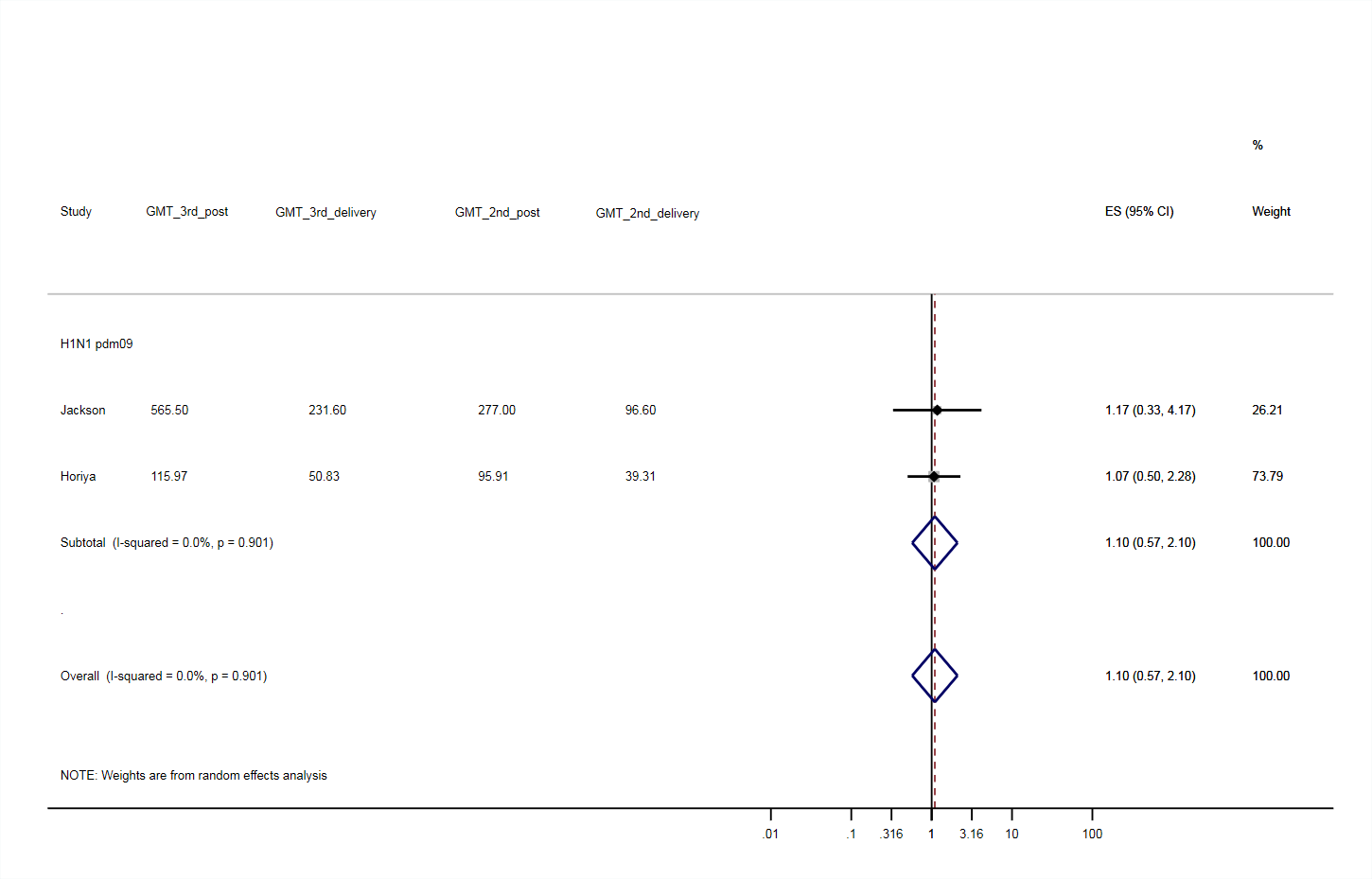


Supplementary Figure 11. A sensitivity analysis that excludes Jackson et al. 49µg group due to risk of internal bias; forest plot of the geometric mean fold decrease (GMFD) ratio, post-2^nd^ immunisation to delivery, comparing women vaccinated in the 3rd trimester with women vaccinated in the 2nd trimester.

GMT_3rd_ post: geometric mean titre (GMT) post-2^nd^ vaccination, 3rd trimester vaccination

GMT_3rd_delivery: GMT in mother at delivery, 3rd trimester vaccination

GMT_2nd_ post: GMT post-2^nd^ vaccination, 2nd trimester vaccination

GMT_2nd_delivery: GMT in mother at delivery, 2nd trimester vaccination

ES (95% CI): Effect size (GMFD) (95% confidence interval)


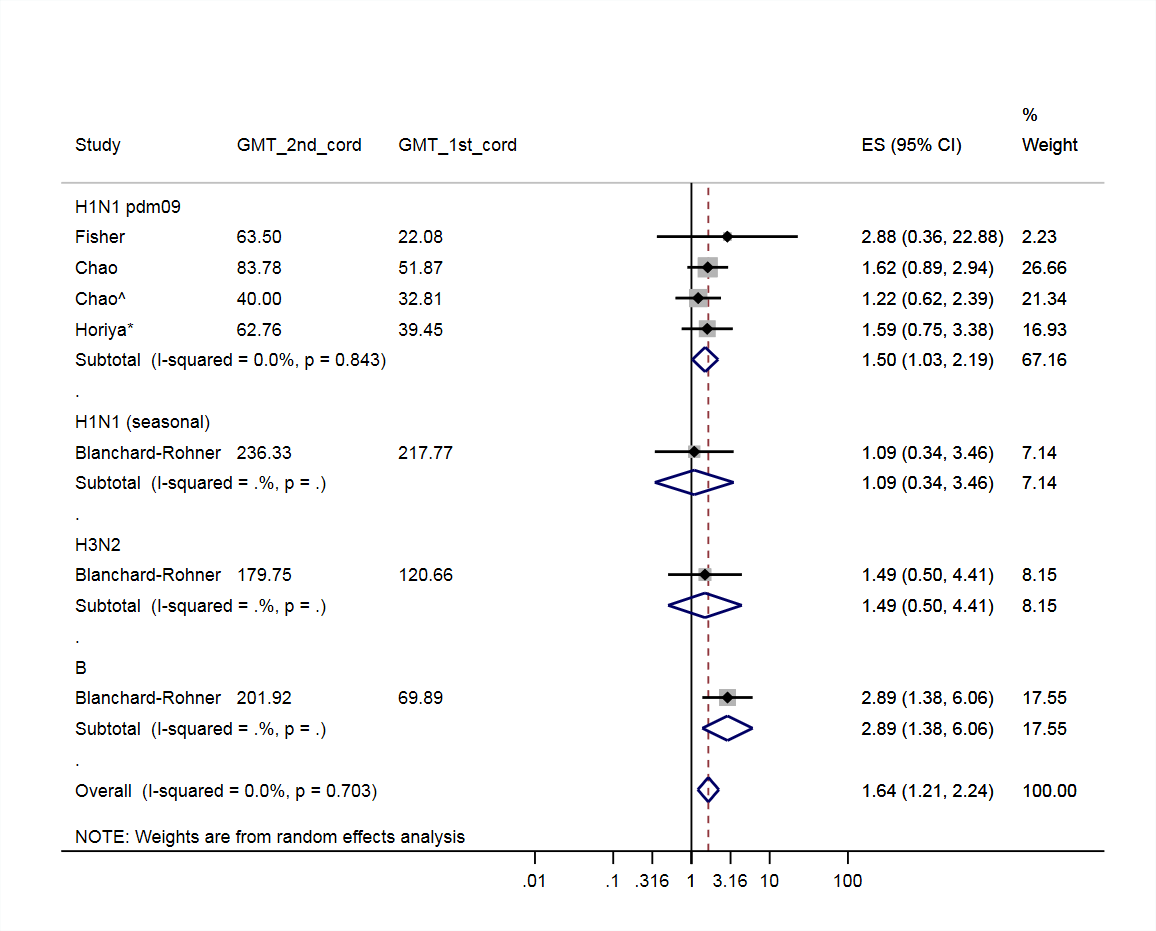


Supplementary Figure 12. A forest plot of the cord-blood GMT ratio (GMR) comparing women vaccinated in the 2^nd^ trimester with women vaccinated in the 1^st^ trimester.

^ Chao et al. adjuvanted vaccine group (as opposed to the un-adjuvanted vaccine group)

* Horiya et al. all women had received two vaccine doses

GMT_2^nd^_cord: geometric mean titre (GMT) in cord-blood at delivery, 2^nd^ trimester vaccination

GMT_1^st^_cord: GMT in cord-blood at delivery, 1^st^ trimester vaccination

ES (95% CI): Effect size (GMR) (95% confidence interval)


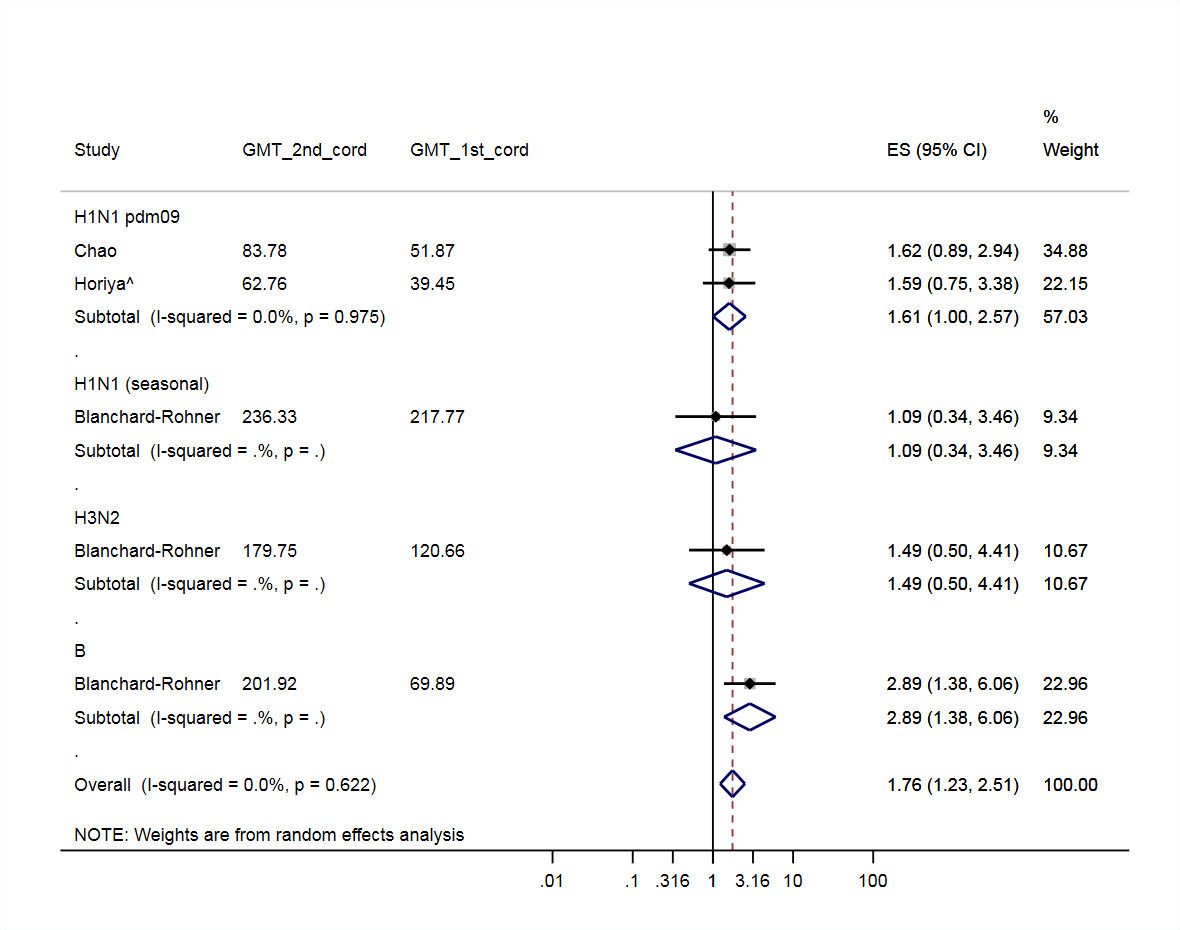


Supplementary Figure 13. A sensitivity analysis that excludes Fisher et al. and Chao et al. adjuvanted group due to risk of internal bias; this is a forest plot of the cord-blood GMT ratio comparing women vaccinated in the 2^nd^ trimester with women vaccinated in the 1^st^ trimester.

^ Horiya et al. all women had received two vaccine doses

GMT_2nd_cord: geometric mean titre (GMT) in cord-blood at delivery, 2nd trimester vaccination

GMT_1st_cord: GMT in cord-blood at delivery, 1st trimester vaccination

ES (95% CI): Effect size (GMR) (95% confidence interval)


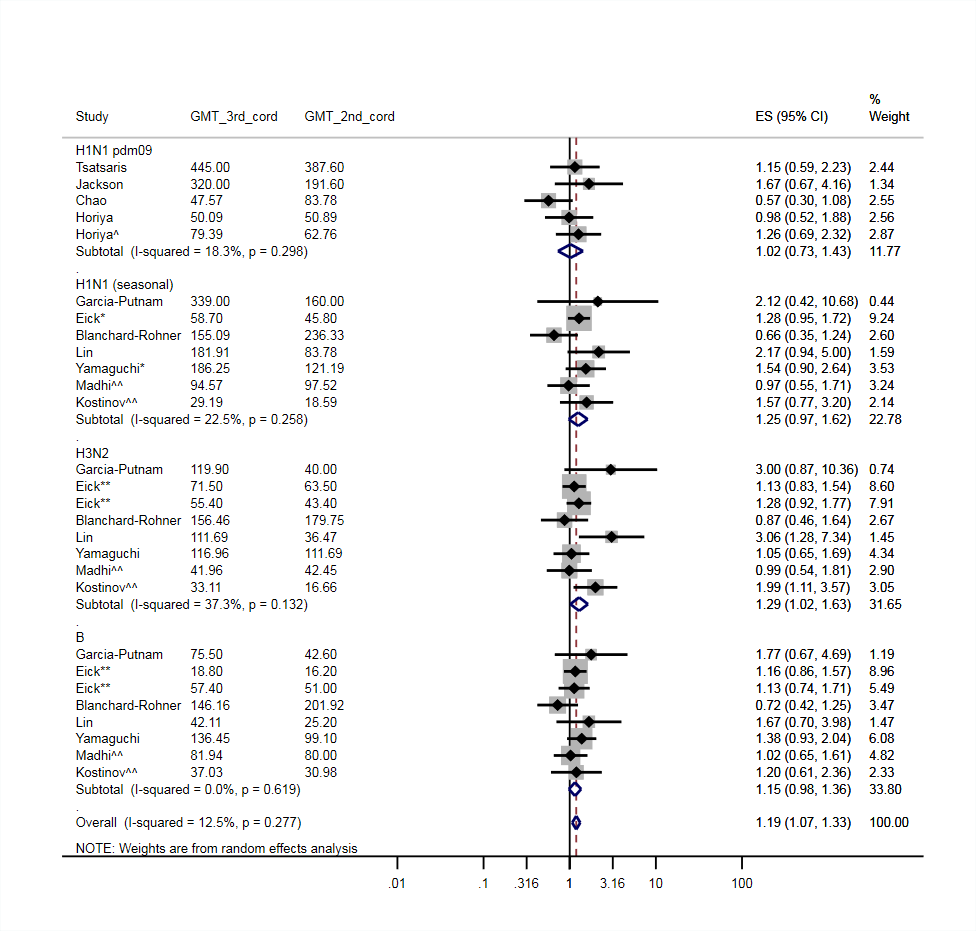


Supplementary Figure 14. A sensitivity analysis that excludes Fisher et al. and Jackson et al. 49mg group due to risk of internal bias; this is a forest plot of the cord-blood GMT ratio comparing women vaccinated in the 3rd trimester with women vaccinated in the 2^nd^ trimester

^ Horiya et al. two vaccine dose group (as opposed to the one vaccine dose group)

^^ Madhi et al. and Kostinov et al. were conducted over two influenza seasons (using same vaccine in both seasons)

* Eick et al. & Yamaguchi et al. studied non-pdm09 H1N1 strain (2002-05 seasons and 2007/08 season, respectively)

** Eick et al. contained different H3N2 and two B strains in two of the three seasons (‘02/’03 & ‘03/’04 vaccines contained the same strains, ‘04/’05 contained different H3N2 & B strains).

GMT_3rd_cord: geometric mean titre (GMT) in cord-blood at delivery, 3rd trimester vaccination

GMT_2nd_cord: GMT in cord-blood at delivery, 2nd trimester vaccination

ES (95% CI): Effect size (GMR) (95% confidence interval)


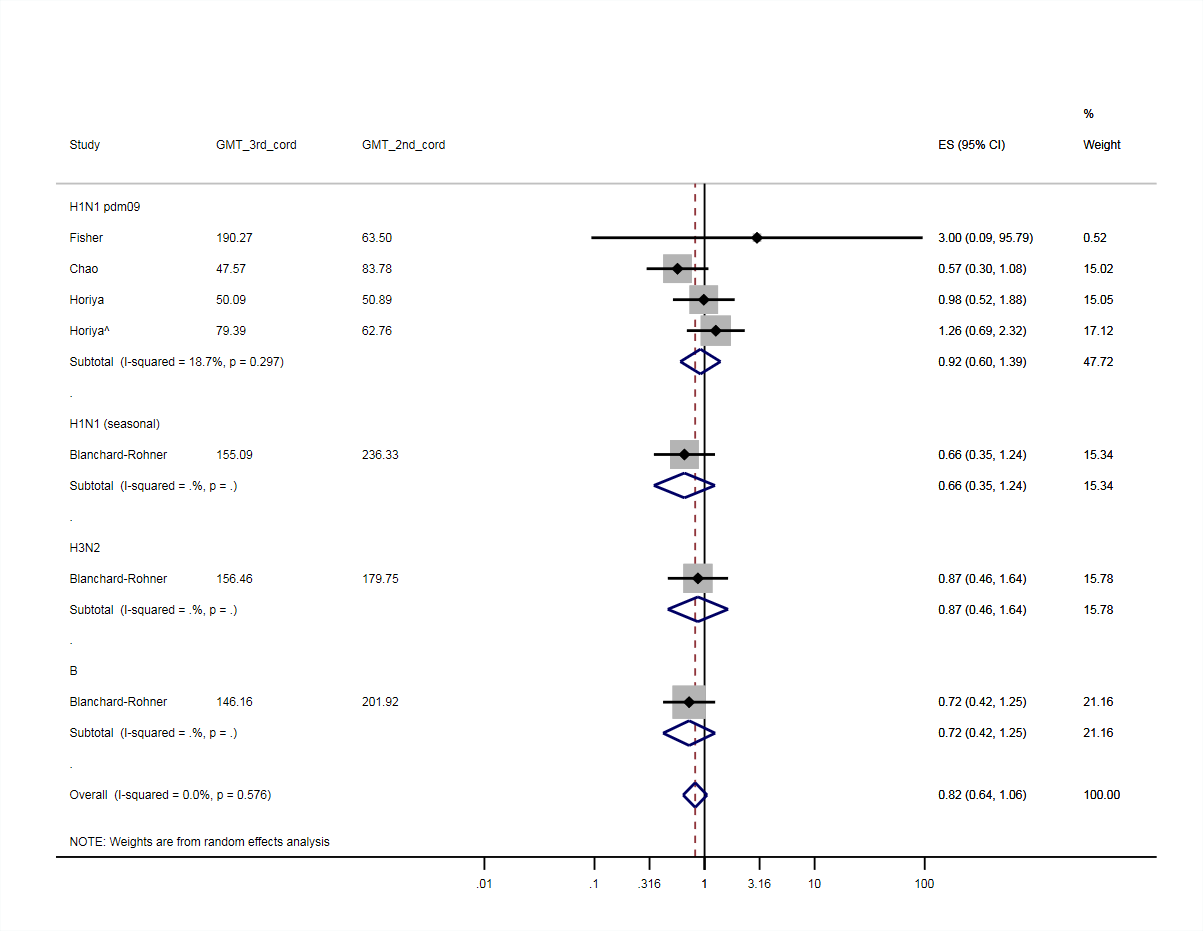


Supplementary Figure 15. A sensitivity analysis including only studies with women vaccinated in the 1^st^ trimester as well the 3^rd^ and 2^nd^ trimesters; this is a forest plot of the cord-blood GMT ratio comparing women vaccinated in the 3rd trimester with women vaccinated in the 2^nd^ trimester.

^ Horiya et al. two vaccine dose group (as opposed to the one vaccine dose group)

GMT_3rd_cord: geometric mean titre (GMT) in cord-blood at delivery, 3rd trimester vaccination

GMT_2nd_cord: GMT in cord-blood at delivery, 2nd trimester vaccination

ES (95% CI): Effect size (GMR) (95% confidence interval)


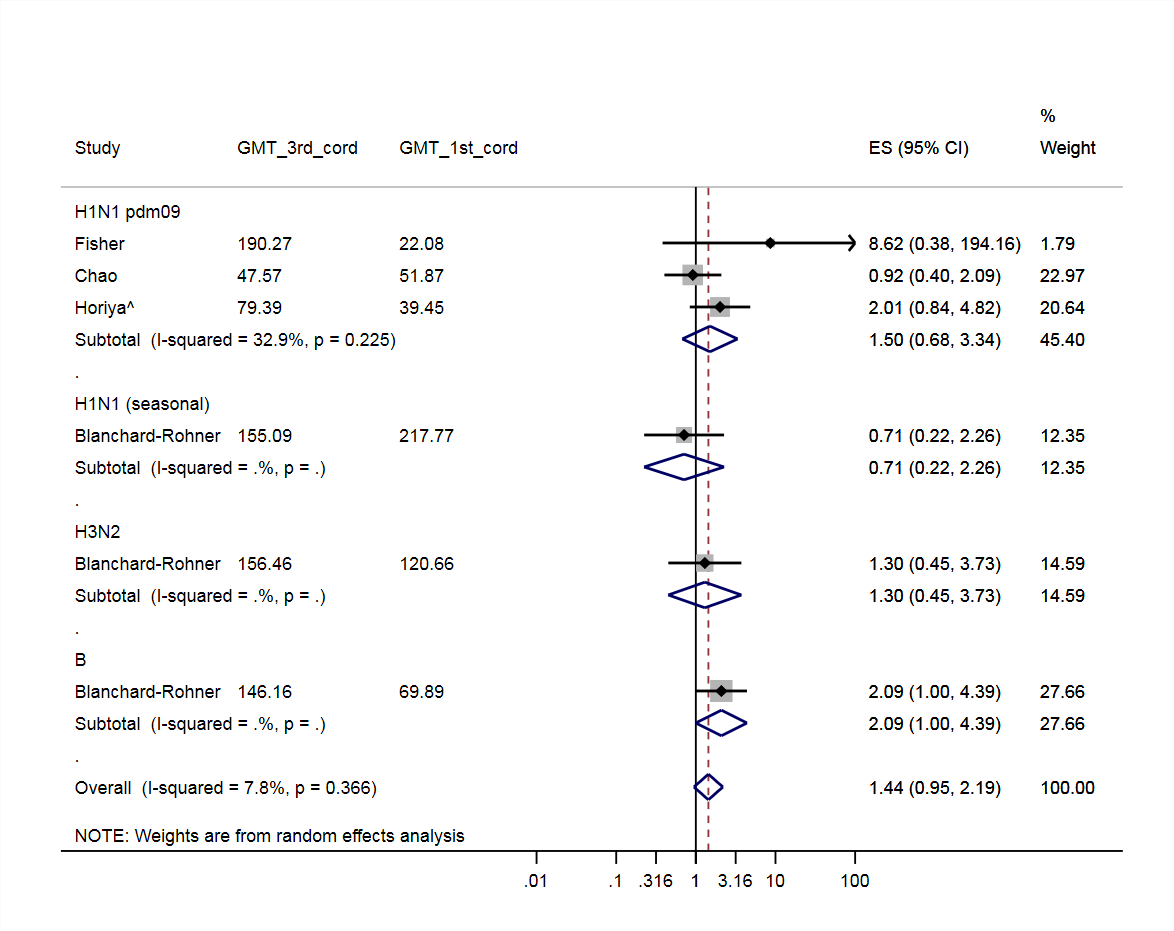


Supplementary Figure 16. A forest plot of the cord-blood GMT ratio comparing women vaccinated in the 3^rd^ trimester with women vaccinated in the 1^st^ trimester.

^ Horiya et al. all women had received two vaccine doses

GMT_3^rd^_cord: geometric mean titre (GMT) in cord-blood at delivery, 3^rd^ trimester vaccination

GMT_1^st^_cord: GMT in cord-blood at delivery, 1^st^ trimester vaccination

ES (95% CI): Effect size (GMR) (95% confidence interval)


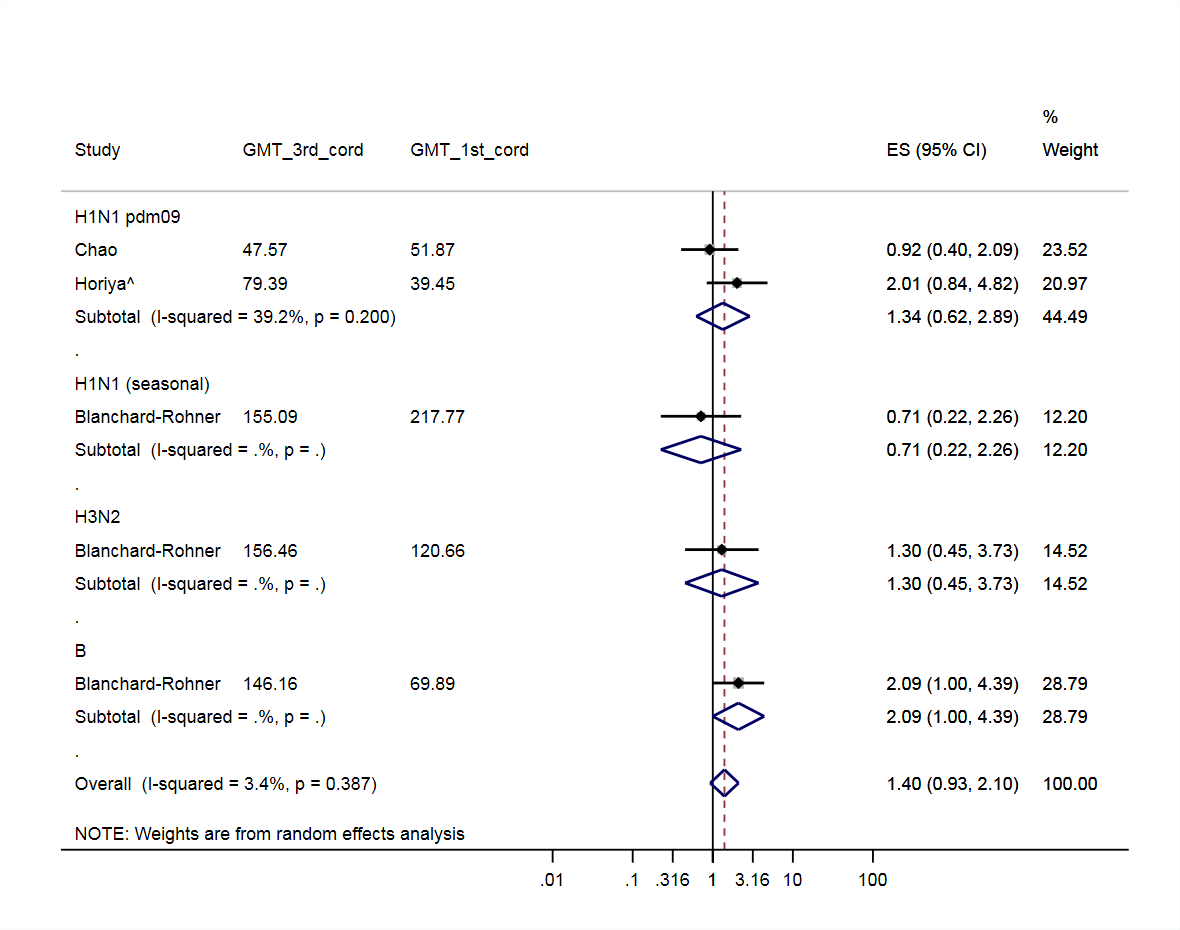


Supplementary Figure 17. A sensitivity analysis that excludes Fisher et al. due to risk of internal bias; this is a forest plot of the cord-blood GMT ratio comparing women vaccinated in the 3rd trimester with women vaccinated in the 1st trimester.

^ Horiya et al. all women had received two vaccine doses

GMT_3rd_cord: geometric mean titre (GMT) in cord-blood at delivery, 3rd trimester vaccination

GMT_1st_cord: GMT in cord-blood at delivery, 1st trimester vaccination

ES (95% CI): Effect size (GMR) (95% confidence interval)
